# Electrolyte Design for Simultaneous Interfacial Stabilization in Si||NCM811 Full Cells

**DOI:** 10.1002/advs.76056

**Published:** 2026-06-09

**Authors:** Seo Yun Jang, Kyu Hong Lee, Juhyoung Kim, Dong Wook Kim, Kyu Hyoung Lee, Do Youb Kim, Se‐Hee Kim

**Affiliations:** ^1^ Advanced Battery Research Center Korea Research Institute of Chemical Technology (KRICT) Daejeon Republic of Korea; ^2^ Department of Chemical and Biomolecular Engineering Yonsei University Seoul Republic of Korea; ^3^ Department of Materials Science and Engineering Yonsei University Seoul Republic of Korea; ^4^ Department of Advanced Materials and Chemical Engineering University of Science and Technology (UST) Daejeon Republic of Korea

**Keywords:** interfacial stability, liquid electrolytes, lithium‐ion full cells, NCM811 cathodes, SEI/CEI engineering, Silicon anodes

## Abstract

The combination of silicon anode and Ni‐rich cathode offers high energy density but suffers from severe interfacial instabilities during cycling. Silicon undergoes repeated volume changes that destabilize its solid electrolyte interphase (SEI), while NCM811 experiences surface degradation and transition‐metal dissolution, both of which are exacerbated by electrolyte‐derived side reactions. Here, a dual‐additive electrolyte design incorporating fluoroethylene carbonate (FEC), a commercially established SEI‐forming additive, and dimethoxydimethylsilane (DMDMS) is proposed to stabilize both electrode interfaces simultaneously. FEC promotes the formation of a LiF‐rich SEI on the silicon anode, whereas DMDMS suppresses acid‐driven degradation by scavenging HF and stabilizing PF_5_. This design effectively mitigates gas evolution, interfacial impedance growth, and electrode swelling. Benefiting from this synergistic electrolyte formulation, Si‐Fe alloy anode‐based Si||NCM811 full cells deliver an initial discharge capacity of 167.8 mAh g^−1^ and retain 65.5% of the capacity after 150 cycles, together with improved rate capability. These results highlight an effective electrolyte formulation strategy for enhancing the durability of high‐energy Si||NCM lithium‐ion batteries.

## Graphical abstract

A dual‐additive electrolyte (DAEL) system was optimized to regulate interfacial reactions in both Si anode and the NCM811 cathode. DAEL suppresses gas evolution and degradation, enabling stable interphases. These synergistic effects enhance electrochemical stability and extend battery cycle life in high‐energy lithium‐ion cells.

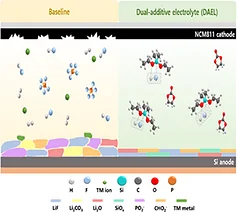

## 1 Introduction

The integration of high‐capacity silicon anodes with Ni‐rich layered oxide cathodes, such as LiNi_0.8_Co_0.1_Mn_0.1_O_2_ (NCM811), is widely regarded as one of the most promising approaches for next‐generation lithium‐ion batteries (LIBs) [1, 2, 3]. Silicon offers an ultrahigh theoretical capacity of ∼ 4200 mAh g^−1^, nearly an order of magnitude higher than that of graphite, while Ni‐rich layered oxides provide high specific capacity with reduced cobalt content, which is desirable from both economic and environmental perspectives [4, 5]. However, the practical integration of these materials in full‐cell configurations introduces severe interfacial challenges. Si anodes undergo drastic volume expansion and contraction of up to ∼ 300% during lithiation and delithiation [6, 7, 8]. These repeated mechanical stresses induce particle cracking, pulverization, and loss of electrical contact, thereby disrupting lithium‐ion transport pathways and accelerating capacity fading [9, 10, 11]. More critically, the large volume fluctuations repeatedly fracture the solid electrolyte interphase (SEI), continuously exposing fresh silicon surfaces to the electrolyte. This process leads to excessive consumption of active lithium and electrolyte, forming a thick and unstable SEI that electrically isolates silicon particles and limits cycle life [6, 11, 12]. Under high‐voltage operation, Ni‐rich NCM811 cathodes suffer from surface reconstruction, oxygen release, and transition‐metal (TM) dissolution [13, 14, 15, 16]. In particular, dissolved Ni^2+^ species migrate through the electrolyte and deposit on the Si anode, where they catalyze electrolyte decomposition and further destabilize the SEI [17, 18, 19, 20]. These degradation processes are strongly aggravated by the intrinsic instability of the commonly used salt LiPF_6_. Even trace amounts of moisture trigger LiPF_6_ hydrolysis, generating HF and POF_3_ [21, 22, 23, 24]. HF accelerates TM dissolution and directly attacks the SEI, while POF_3_ and related fluorophosphate intermediates promote solvent decomposition [21, 25, 26]. Under full‐cell conditions, these parasitic reactions accumulate, leading to gas evolution (e.g., H_2_), increased internal pressure, and compromised cell safety [27, 28]. In addition, various electrode‐level approaches, such as surface sheath/coating strategies and functional polymeric binders, have also been explored to suppress interfacial side reactions and stabilize Si‐based electrodes [29, 30, 31]. From the electrolyte perspective, extensive efforts have focused on the use of functional electrolyte additives to regulate interfacial reactions and improve electrolyte stability. Electrolyte additive engineering has therefore emerged as an effective approach to mitigate these coupled interfacial degradation processes [32]. Fluoroethylene carbonate (FEC) is a commercially established additive for silicon‐based anodes, as it preferentially decomposes to form LiF‐rich SEI layers with enhanced mechanical robustness [33, 34]. However, in practical full‐cell systems, FEC alone is insufficient. Under high‐voltage or elevated‐temperature conditions, FEC decomposition can generate HF and H_2_, which further aggravate interfacial instability [35]. As a result, while FEC improves SEI formation on silicon anodes, it does not adequately suppress HF‐mediated degradation at the cathode‐electrolyte interphase (CEI). To address these limitations, we propose a dual‐additive electrolyte formulation that combines FEC with DMDMS. In this study, the Baseline electrolyte is defined as 1.15 M LiPF_6_ in a 2:4:4 (v/v) mixture of ethylene carbonate (EC), dimethyl carbonate (DMC), and ethyl methyl carbonate (EMC). The FEC‐containing electrolyte is referred to as the single‐additive electrolyte (SAEL), whereas the FEC/DMDMS formulation is denoted as the dual‐additive electrolyte (DAEL). Using Si‐Fe alloy‐based Si anodes paired with NCM811 cathodes, we systematically investigate the electrochemical performances, interfacial chemistry, and degradation mechanisms of Si||NCM811 full cells employing the Baseline, SAEL, and DAEL formulations.

## 2 Results and Discussion

**FIGURE 1:**
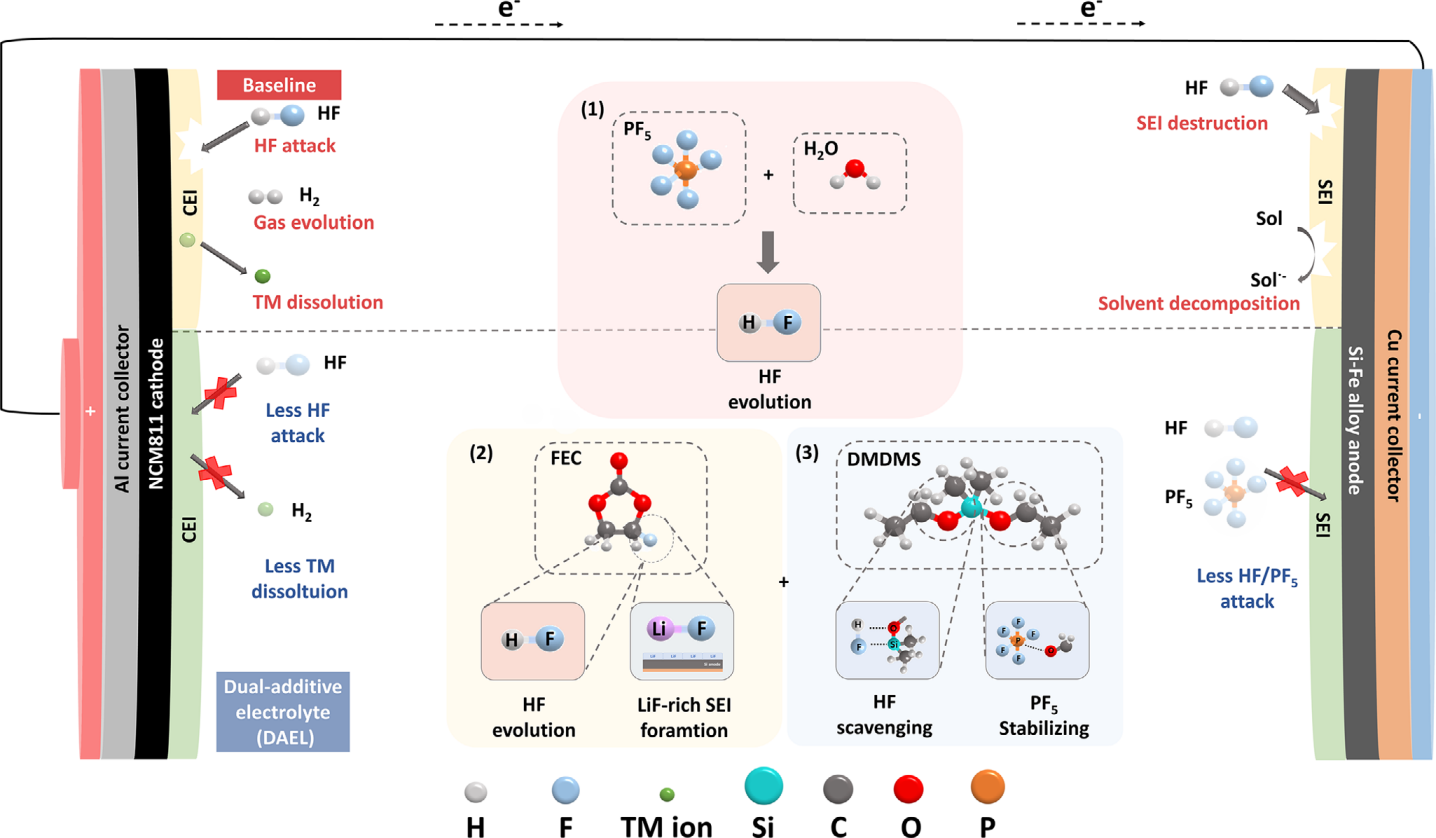
Electrolyte design concept and degradation mitigation mechanism.

Figure 1 illustrates the key degradation pathways in Si||NCM811 full cells and the corresponding mitigation strategy enabled by DAEL. In conventional Baseline electrolytes, repeated volume changes of silicon anodes during cycling lead to continuous fracture and reformation of the SEI, resulting in active lithium loss and accelerated capacity fading. Concurrently, the commonly used salt LiPF_6_ undergoes thermal and hydrolytic decomposition, forming PF_5_, which further reacts with trace H_2_O to generate HF and POF_3_ (as depicted in (1) of Figure 1) [36]. These acidic species promote TM dissolution from the NCM811 cathode, particularly Ni^2+^, and catalyze electrolyte decomposition accompanied by gas evolution, thereby undermining long‐term cell stability. In DAEL, the incorporation of DMDMS into FEC‐containing electrolytes suppresses these coupled degradation processes by scavenging HF and stabilizing PF_5_ [37]. The silane‐based reactivity of DMDMS enables chemical neutralization of HF, while its interaction with PF_5_ mitigates autocatalytic electrolyte decomposition (2). As a result, DAEL facilitates the formation of LiF‐rich and chemically robust SEI and CEI layers, reduces TM dissolution, and enhances interfacial stability under high‐voltage operation (3). In particular, HF‐induced leaching of interfacial species and extraction of Ni^2+^ ions, which lead to the loss of active lithium storage sites, are effectively suppressed [38].

### 2.1 Electrolyte Design Rationale and Physicochemical Properties

**FIGURE 2:**
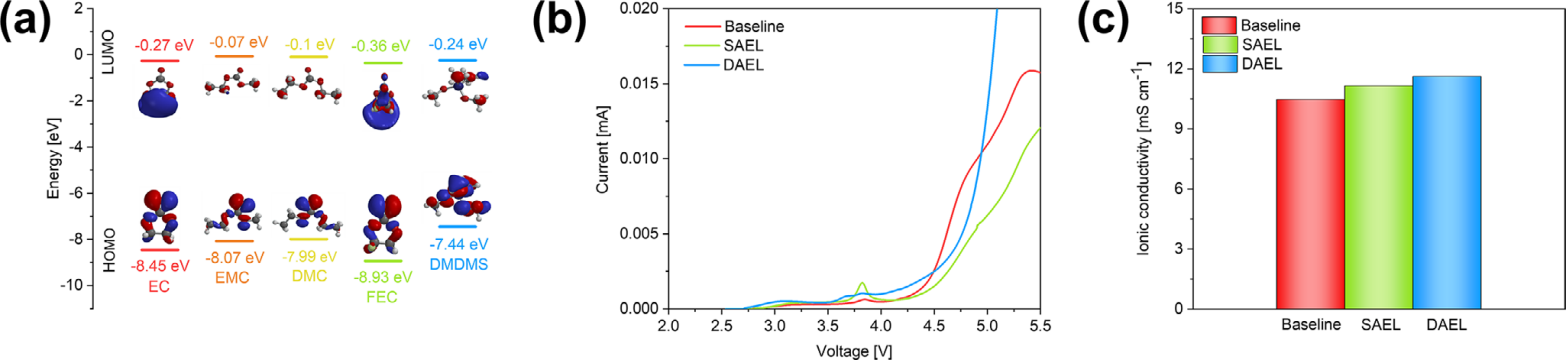
Electrochemical and physicochemical properties of electrolytes, (a) HOMO and LUMO energy levels and orbital distributions of electrolyte components (EC, EMC, DMC, FEC, and DMDMS), (b) Linear sweep voltammetry curves showing oxidative stability with different additive combinations using Li||SUS cell, (c) Ionic conductivity of electrolytes with different additives.

To rationalize the design concept of the DAEL and establish a basis for interpreting subsequent electrochemical results, the intrinsic electrochemical characteristics of the additives were first examined (Figure 2). Frontier orbital energy calculations were employed to evaluate the preferential decomposition behavior of each component at the electrode interfaces. As shown in Figure 2a, DMDMS exhibits the highest HOMO energy level (−7.44 eV) among the solvents, indicating a stronger propensity for oxidative decomposition at the cathode. In contrast, its relatively low LUMO energy level (−0.24 eV) suggests a limited tendency toward reductive decomposition at the anode, thereby mitigating excessive reduction‐driven side reactions. These electronic features imply that DMDMS preferentially participates in cathode‐side interfacial reactions, contributing to the formation of a stable CEI [39, 40]. The electrochemical stability of the electrolytes was further evaluated by linear sweep voltammetry (Figure 2b). The Baseline, SAEL, and DAEL formulations exhibit comparable oxidative stability, with no pronounced differences observed up to approximately 4.3 V. Although minor variations in the onset of oxidative current are detected, all electrolytes maintain similar electrochemical stability windows within this voltage range, indicating their electrochemical stability within the practical operating voltage range of conventional full‐cell systems. Cyclic voltammetry (CV) measurements using Li||SUS cells (Figure S1) reveal improved electrochemical reversibility for DAEL, as evidenced by more consistent and overlapping current responses over successive cycles compared to the Baseline and SAEL. This behavior suggests improved electrochemical stability and more stable redox processes. In addition, ionic conductivity measurements (Figure 2c and Figure S2) show that DAEL achieves the highest bulk ionic conductivity among the various formulations. Specifically, the ionic conductivities of the Baseline electrolyte, SAEL, and DAEL are measured to be 10.03, 10.72, and 11.62 mS cm^−1^, respectively, with DAEL exhibiting the highest value. The enhanced ionic transport in DAEL is attributed to the formation of Si–O–Si crosslinked structures, which facilitate lithium‐ion solvation and transport [41]. Moreover, the incorporation of DMDMS reduces the surface tension of the electrolyte, as reflected by a decrease in the contact angle on the separator from 37.5 ° for SAEL to 32.2 ° for DAEL (Figure S3). These results demonstrate that the DAEL improves interfacial wettability and ionic conductivity while maintaining sufficient oxidative stability, providing a favorable physicochemical foundation for subsequent electrochemical performance.

### 2.2 Regulation of Cathode‐Side Interfacial Chemistry in DAEL System

**FIGURE 3:**
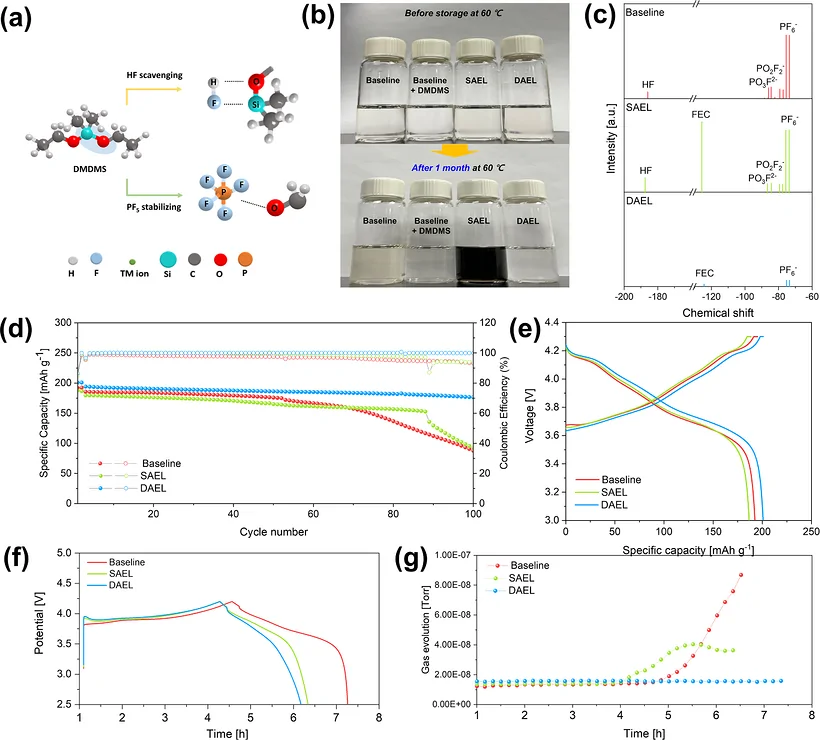
Electrolyte formulation strategy for interfacial stabilization and gas suppression for high‐performance NCM811 cells, (a) Dual role of DMDMS in HF scavenging and PF_5_ stabilization, (b) Visual changes in electrolytes after one‐month storage at 60°C, (c) ^19^F NMR spectra showing HF removal by DMDMS, (d) Cycling performance and Coulombic efficiency of NCM811 half‐cells at 0.5C between 3.0 and 4.3 V, (e) Voltage–capacity profiles of NCM811 half‐cells during initial cycles, (f) Potential profiles and (g) H_2_ evolution behavior over time for different electrolytes during initial charging and discharging.

To elucidate the role of DAEL in regulating cathode‐side interfacial chemistry, chemical and electrochemical analyses were performed (Figure 3). DMDMS, a key constituent of DAEL, contains siloxane groups capable of capturing HF generated from LiPF_6_ decomposition, thereby mitigating proton‐driven interfacial degradation. Furthermore, its Lewis‐basic Si–O‐Si moieties can coordinate with PF_5_, reducing the salt's reactivity at the Ni‐rich cathode surface [39, 40, 42]. As illustrated in Figure 3a, these stabilization effects proceed through two complementary pathways: (i) proton binding at Si–O sites followed by the formation of stable Si–F species, enabling efficient HF removal and (ii) nucleophilic reactions between PF_5_ and methoxy groups or the silicon center, which suppress the formation of POF_3_ and related fluorophosphate byproducts [36, 38, 41, 43, 44, 45, 46]. Through these processes, the concentration of acidic species is reduced, thereby stabilizing the CEI and suppressing chain decomposition reactions. The effectiveness of DMDMS in improving thermal stability was further evaluated by storage tests at 60°C for 30 days (Figure 3b). While SAEL exhibited pronounced discoloration, DAEL retained optical clarity [47, 48]. This behavior is consistent with the thermal reactivity of FEC toward PF_5_ at elevated temperatures, which promotes the formation of HF and fluorophosphate intermediates in LiPF_6_‐based electrolytes [49]. The suppressed discoloration of DAEL indicates that DMDMS retards thermally induced decomposition. This enhanced stability was also evident during earlier storage stages (5 days and 2 weeks), where the DMDMS‐containing electrolytes remained largely transparent (Figure S4). To directly probe the HF scavenging behavior of DMDMS, ^19^F NMR spectroscopy was conducted after storing the electrolytes with 500 ppm deionized water for three days (Figure 3c). Additional electrochemical results for the Baseline + DMDMS electrolyte are provided in Figure S5, further supporting the independent interfacial stabilization effect of DMDMS. Strong HF signals were detected for SAEL, reflecting LiPF_6_ hydrolysis in the presence of trace moisture [50]. In contrast, HF signals were negligible for DAEL, confirming that DMDMS effectively suppresses HF accumulation under water‐added conditions. This result indicates that DMDMS not only captures existing HF but also limits further HF formation by stabilizing PF_5_. The cathode‐side benefits of HF and PF_5_ suppression were further reflected in the electrochemical performance of NCM811 half‐cells. After 100 cycles, the Baseline and SAEL electrolytes exhibited pronounced capacity decay, decreasing from 185.6 to 81.4 mAh g^−1^ and from 179.4 to 88.4 mAh g^−1^, corresponding to capacity retentions of 43.9% and 49.3%, respectively. In contrast, DAEL showed significantly improved cycling stability, maintaining a capacity of 175.0 mAh g^−1^ from an initial value of 194.1 mAh g^−1^, yielding a high retention of 90.1% (Figure 3d,e) [36, 51]. This improvement is primarily attributed to the suppression of acid‐induced interfacial degradation at the Si anode, enabled by DMDMS‐mediated HF mitigation. In situ Differential Electrochemical Mass Spectrometry (DEMS) measurements provide direct evidence of suppressed gas evolution in DAEL under galvanostatic cycling at 0.3C with an areal capacity of 2.6 mAh cm^−2^ (Figure 3f,g) [52]. While Baseline and SAEL electrolytes exhibit substantial H_2_ evolution during charging, these signals are markedly reduced in DAEL. This behavior reflects the effective suppression of HF‐induced reactions and PF_5_‐mediated electrolyte decomposition by DMDMS [38, 53]. H_2_ gas, typically originating from the electrochemical reduction of HF or high‐voltage solvent decomposition, accelerates electrolyte degradation and interfacial instability [54]. The reduced discharge capacity observed under DEMS conditions, relative to conventional half‐cell cycling, is attributed to the confined electrolyte volume and enhanced sensitivity of DEMS to parasitic reactions, which amplify side reactions such as HF‐induced decomposition and lead to pronounced lithium consumption [46, 55]. Consequently, the suppression of H_2_ evolution in DAEL highlights the critical role of DMDMS in stabilizing cathode‐side interfacial chemistry and mitigating PF_5_‐driven degradation pathways [38].

### 2.3 Anode‐Side Stabilization in DAEL System

**FIGURE 4:**
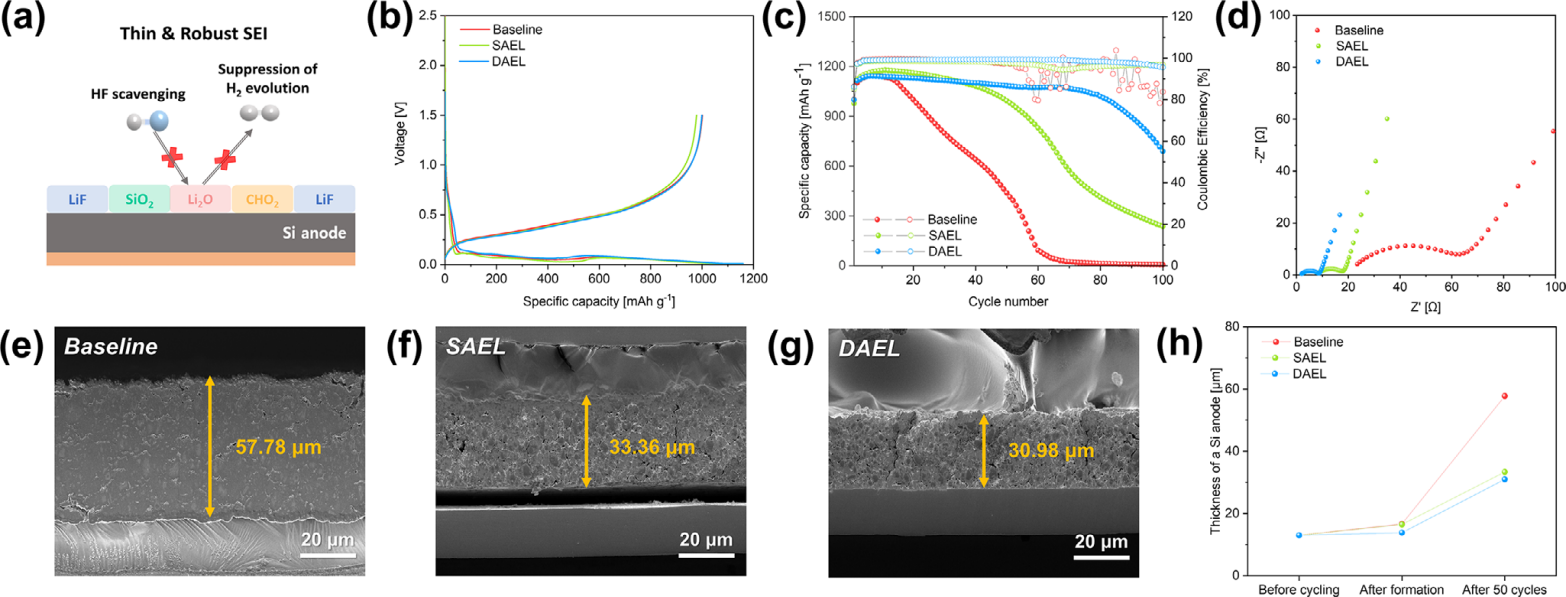
Electrolyte‐dependent interfacial stabilization and structural evolution in Si half‐cells, (a) SEI formation mechanism involving HF scavenging and electrolyte decomposition, (b) Voltage–capacity profiles of Si half‐cells during initial cycling. (c) Cycling performance and Coulombic efficiency of Si half‐cells at 0.5C between 0.01 and 1.5 V, (d) EIS spectra after 100 cycles in Si half‐cells. Cross‐sectional SEM images of Si electrodes after 50 cycles in (e) Baseline, (f) SAEL and (g) DAEL with different electrolytes, (h) Electrode thickness changes before cycling, after formation, and after 50 cycles.

The influence of the DAEL on Si anodes was systematically investigated through electrochemical evaluation, interfacial impedance analysis, and post‐cycling structural, chemical, and morphological characterization. This section correlates electrochemical performance with the chemical composition and physical integrity of the SEI, providing a comprehensive understanding of anode‐side interfacial stabilization enabled by the DAEL formulation. Figure 4a schematically illustrates the proposed SEI formation mechanism, in which the suppression of HF‐driven reactions limits H_2_ evolution and electrolyte decomposition, thereby promoting the formation of a thinner and more stable SEI. The electrochemical performance of Si half‐cells with different electrolyte formulations is compared in Figure 4b,d. After 50 cycles, the Baseline electrolyte shows significant capacity fading, decreasing from 1121.8 to 393.1 mAh g^−1^, corresponding to a capacity retention of 35%, indicating pronounced interfacial instability. SAEL exhibits improved stability, retaining 85.1% of its initial capacity (1136.9 → 967.8 mAh g^−1^). Remarkably, DAEL demonstrates superior performance with capacity increasing from 1123.6 to 1080.1 mAh g^−1^, achieving a retention of 96.1%, underscoring its excellent interfacial stabilization effect on the Si anode. CV measurements using Si||Li half‐cells (Figure S6) further support these trends, with the DAEL exhibiting the most stable and overlapping redox peaks over repeated cycles, suggesting enhanced reversibility of lithiation and delithiation and suppressed interfacial side reactions. Due to the large volume changes of silicon during lithiation, Si anodes typically undergo particle pulverization and continuous SEI reformation in liquid electrolytes, leading to severe loss of lithium inventory [11]. This effect is further exacerbated in half‐cell configurations by the effectively unlimited lithium supply from the lithium metal counter electrode [56]. In the Baseline electrolyte, these degradation processes are amplified by unstable SEI formation and continuous electrolyte decomposition, resulting in rapid capacity decay. Electrochemical impedance spectroscopy (EIS) (Figure 4d) reveals that the charge‐transfer resistance (R_ct_) decreases sequentially from the Baseline electrolyte to SAEL and further to the DAEL after 100 cycles, indicating progressively improved interfacial stability. Time‐resolved impedance analysis (Figure S7) confirms that the DAEL maintains the lowest impedance throughout prolonged cycling, consistent with the formation of a stable and well‐preserved interphase. Cross‐sectional Scanning Electron Microscope (SEM) images obtained after 50 cycles (Figure 4e–g and Figure S8) further reveal pronounced differences in electrode swelling behavior. While the pristine Si electrode exhibits an initial thickness of approximately 13.4 µm, severe expansion to 57.78 µm is observed in the Baseline cell. In contrast, electrodes cycled with SAEL and the DAEL show reduced thicknesses of 33.36 and 30.98 µm, respectively. Quantitative analysis of electrode thickness evolution before cycling, after formation, and after 50 cycles (Figure 4h) confirms that the DAEL most effectively mitigates volume expansion during repeated lithiation and delithiation, facilitating mechanically stable SEI formation [2, 11].

**FIGURE 5:**
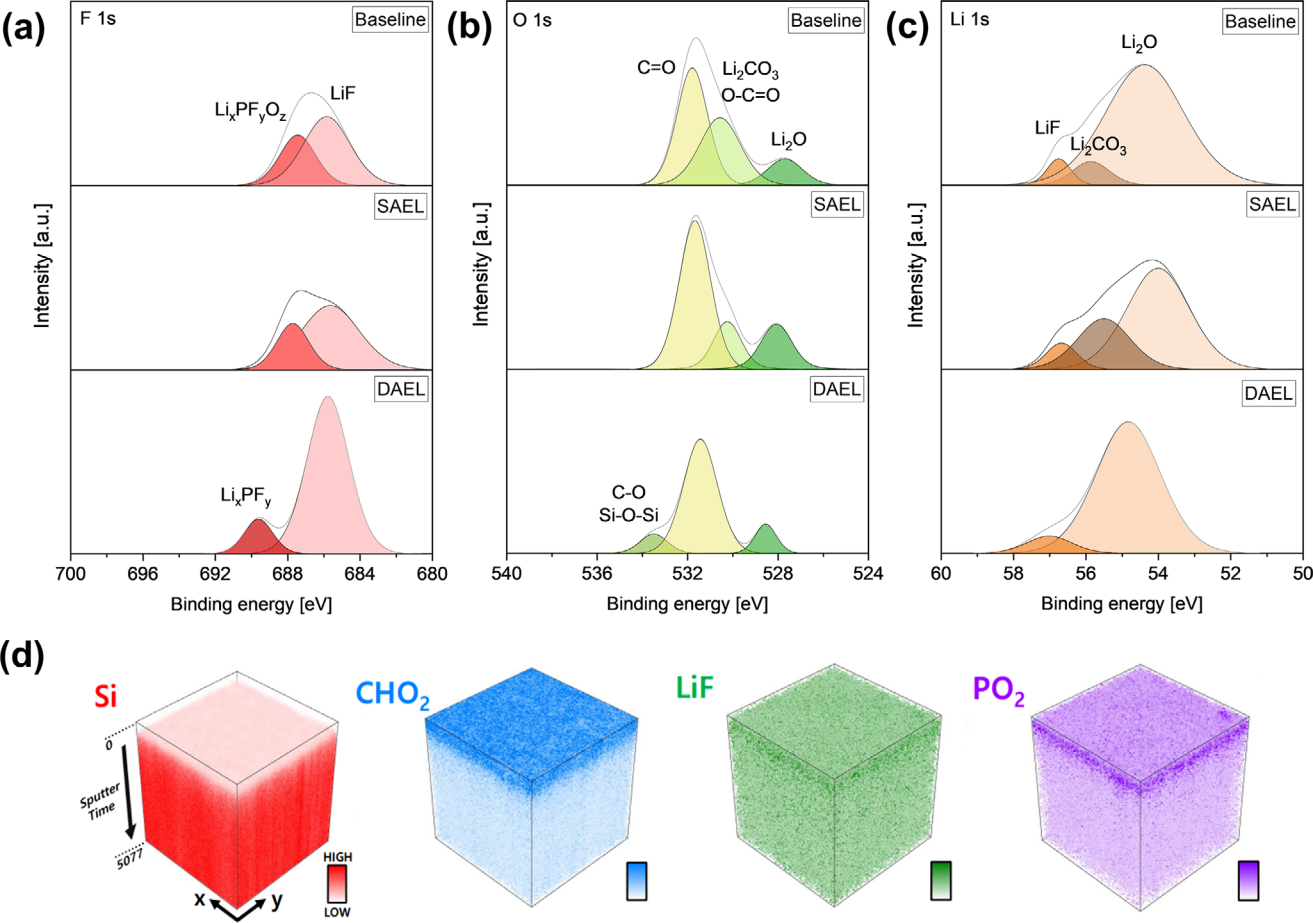
SEI compositional and structural analysis revealing enhanced inorganic‐rich and uniform interphase formation with DAEL, (a) XPS F 1s peaks after 50 cycles, indicating SEI composition, (b) XPS O 1s peaks after 50 cycles, indicating SEI composition, (c) XPS Li 1s peaks after 50 cycles, indicating SEI composition, (d) TOF‐SIMS depth profiles after 50 cycles for DAEL.

To elucidate the chemical origin of the enhanced interfacial stability, the composition and depth‐dependent structure of the SEI were further examined using spectroscopic techniques, including X‐ray photoelectron spectroscopy (XPS) and 3D time‐of‐flight secondary ion mass spectrometry (ToF‐SIMS) (Figure 5). XPS analyses (Figure 5a–c) show that the SEI formed with the DAEL is enriched with inorganic components, particularly LiF and Li_2_O, as evidenced by intensified F 1s and Li 1s signals. A distinct LiF peak near 685 eV becomes increasingly prominent with argon‐ion sputtering depth (Figure 5a,d,e), while the Li_2_O component is observed at approximately 54 eV in the Li 1s spectrum, confirming the dominance of inorganic lithium‐containing species. LiF‐rich SEI structures are known to exhibit enhanced mechanical strength and chemical stability [41]. The pronounced peak shifts observed in the F 1s and Li 1s spectra can be attributed to significant alterations in the chemical composition and local bonding environments within the interphase. Specifically, the DAEL system promotes the formation of a LiF‐rich interphase while effectively suppressing the evolution of Li_x_PF_y_O_z_ and organic decomposition products. This compositional transition toward a more uniform, inorganic‐dominated interphase modifies the electronic environment surrounding the Li and F species, thereby resulting in the observed peak shifts. The O 1s spectrum further reveals a pronounced Si–O‐Si signal centered at approximately 533 eV (Figure 5b), which is more prominent for the DAEL [57]. Such Si─O─Si bonding is considered beneficial, as it provides mechanical flexibility and buffers stress during silicon volume changes, thereby suppressing continuous side reactions [41]. The resulting Li_2_O–LiF composite SEI is mechanically robust yet structurally adaptive, enabling the preservation of interfacial integrity during cycling [58]. 3D ToF‐SIMS elemental mapping (Figure 5d and Figure S9) further corroborates these findings by revealing pronounced compositional differences between the Baseline and DAEL. The DAEL exhibits substantially lower intensities of CHO_2_^−^ and PO_2_^−^ fragments, indicating suppressed decomposition of both organic carbonate solvents and the LiPF_6_ salt [7]. In addition, a more continuous and intense Si signal suggests the formation of a thinner and more uniform SEI layer that allows deeper ion‐beam penetration. In contrast, the Baseline electrolyte exhibits a thick and heterogeneous SEI, characterized by attenuated Si signals and elevated CHO_2_^−^ and PO_2_^−^ intensities along the depth axis, indicative of extensive interfacial degradation [56, 59, 60]. The Baseline and SAEL system show partial improvement but still retains a relatively disordered SEI structure (Figure S10) [58, 61].

**FIGURE 6:**
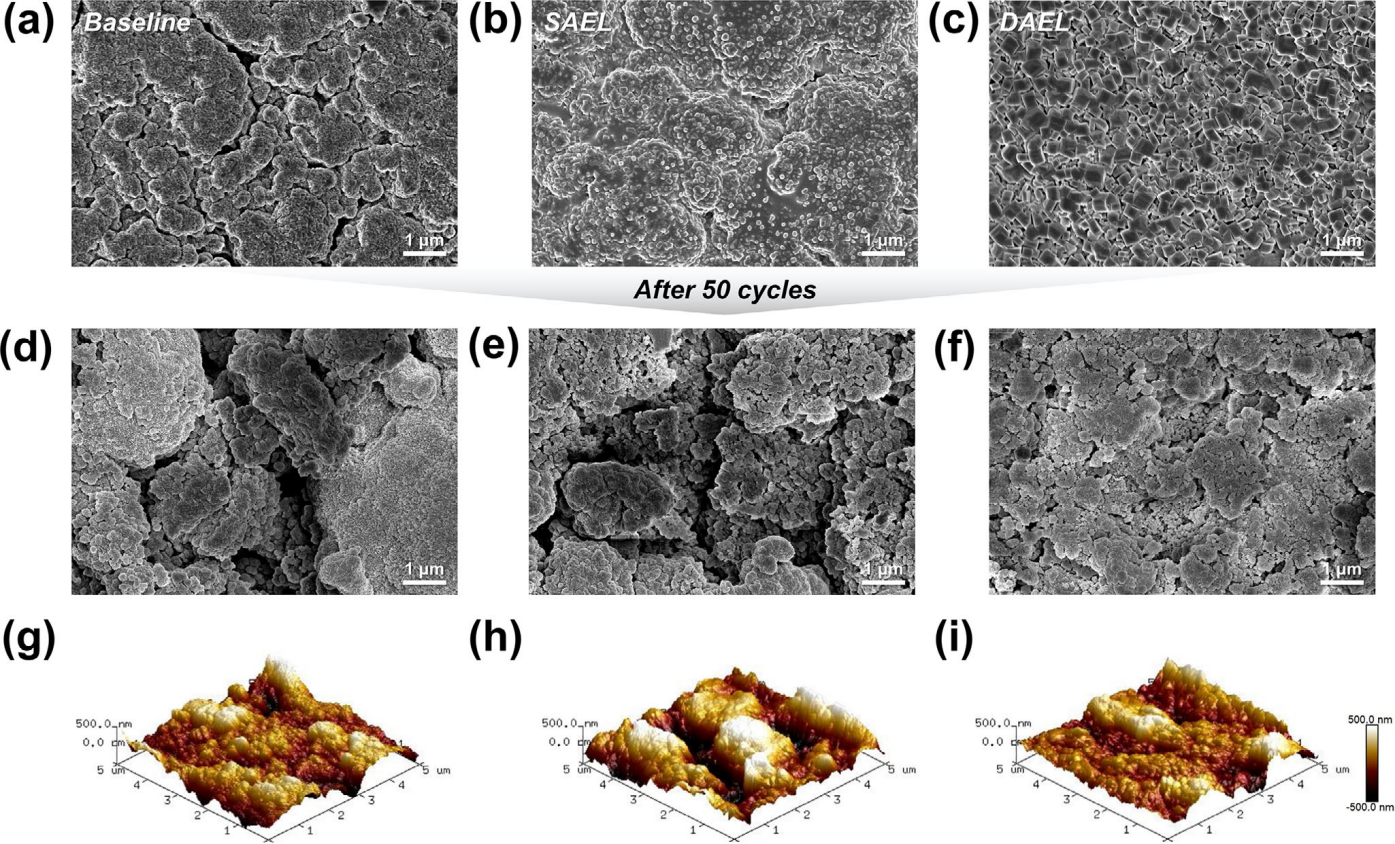
Surface morphological and topographic evolution of Si electrodes during cycling. SEM images of Si electrode surfaces after formation cycle in (a) Baseline, (b) SAEL, and (c) DAEL, and after 50 cycles using (d) Baseline, (e) SAEL, and (f) DAEL. AFM topography images of the corresponding Si electrodes after 50 cycles using (g) Baseline, (h) SAEL, and (i) DAEL.

To further correlate the electrochemical and chemical stabilization effects with the physical integrity of the interphase, surface and morphological characterizations were conducted, as shown in Figure 6. After formation, SEM images reveal severe cracking and non‐uniform surface features for the Baseline electrolyte, indicative of unstable early‐stage SEI formation [62]. The SAEL system shows partial improvement, whereas the DAEL yields a uniform and crack‐free morphology, suggesting effective initial interfacial stabilization (Figure 6a–c). Consistent trends are observed in large‐area and cross‐sectional SEM analyses (Figure S11), where the DAEL forms the thinnest and most compact SEI with markedly fewer cracks and voids, indicating resistance to fracture and suppressed continuous electrolyte decomposition [44, 63, 64]. After 50 cycles, the morphological differences become more pronounced (Figure 6d–f). The Baseline electrode exhibits severe particle agglomeration and extensive surface degradation, while SAEL partially mitigates agglomeration but still shows notable structural damage. In contrast, the electrode cycled with the DAEL maintains a dense and compact surface with minimal degradation, indicating sustained interfacial stability during prolonged cycling. Atomic Force Microscopy (AFM) analyses further support these observations. AFM topographies obtained after formation and after 50 cycles (Figure 6g–i and Figures S12 and S13 and Table S1) show that the DAEL consistently exhibits the lowest surface roughness and a uniform nanoscale topology, whereas the Baseline and SAEL systems develop progressively rougher and more irregular surfaces [63]. Quantitatively, the surface roughness (S_a_) values after formation are 122 nm for the Baseline electrolyte, 170 nm for SAEL, and 95.6 nm for the DAEL (Table S1). After 50 cycles, this trend persists (Baseline: 135 nm, SAEL: 201 nm, DAEL: 121 nm), demonstrating superior long‐term interfacial stability. Corresponding three‐dimensional AFM height maps (Figure S13) further confirm that the DAEL maintains a uniform and compact surface morphology, while reduced S_q_, S_z_, and more negative S_sk_ values indicate suppressed height fluctuations and a plateau‐like topology favorable for mechanically stable SEI formation [65].

### 2.4 Full‐Cell Performance and Interfacial Durability of DAEL System

**FIGURE 7:**
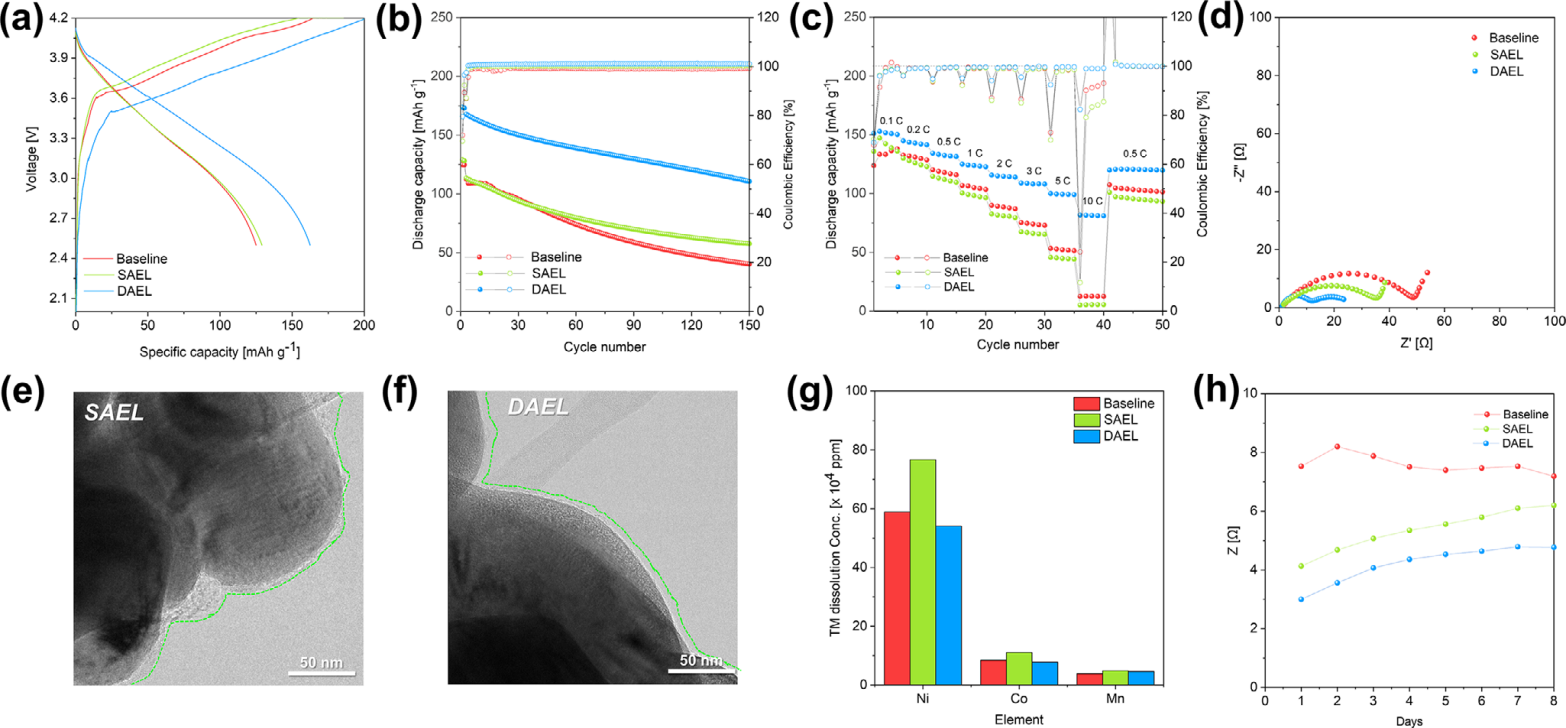
Electrolyte‐enabled enhancement of electrochemical performance and interfacial stability in Si||NCM811 full cells, (a) Voltage profiles during formation, (b) Cycling performance of Si||NCM811 full cells at 0.5 C between 2.5 and 4.2 V, (c) Rate performance of full cells, (d) EIS spectra after 50 cycles in Si||NCM811 full cells. Cs‐TEM images of NCM811 cathodes extracted from Si||NCM811 full cells after 50 cycles with (e) SAEL and (f) DAEL, (g) ICP‐MS analysis of transition metal dissolution from NCM811 cathodes after 50 cycles in Si||NCM811 full cells, (h) Calendar life testing of Si symmetric full cells at 60°C for 8 days.

The improvements observed in half‐cell studies translate directly into enhanced full‐cell performance. As shown in Figure 7a,b, Si||NCM811 full cells employing the DAEL deliver an initial discharge capacity of 167.8 mAh g^−1^ and maintain 109.9 mAh g^−1^ after 150 cycles, corresponding to a capacity retention of 65.5% at an areal loading of 8.9 mg cm^−2^. In contrast, cells using the Baseline electrolyte and SAEL exhibit lower retentions of 35.4% (112.6 to 39.9 mAh g^−1^) and 50.5% (113.5 to 57.3 mAh g^−1^), respectively. This enhanced cycling stability arises from the synergistic interfacial stabilization enabled by the dual‐additive formulation. Additional full‐cell results for the Baseline + DMDMS electrolyte are provided in Figure S14, further validating this synergistic behavior. On the cathode side, acidic species generated from LiPF_6_ decomposition are effectively suppressed, mitigating interfacial degradation of NCM811 under a high charging voltage of 4.2 V. On the anode side, DAEL promotes the formation of a thinner and more uniform SEI while minimizing the accumulation of organic and salt‐derived byproducts. Consequently, structural degradation such as particle agglomeration and surface roughening is significantly mitigated, leading to a denser and more compact electrode morphology. Similar morphology trends were also observed for the Si anodes retrieved from Si||NCM811 full cells after 30 and 50 cycles (Figure S15). These combined effects reduce interfacial resistance and enhance the long‐term electrochemical stability of both electrodes. Rate‐capability tests further highlight these advantages (Figure 7c). Across current densities ranging from 0.1C to 10C, the DAEL consistently delivers higher discharge capacities than SAEL. Upon returning to 0.5C, the cell recovers nearly its capacity, indicating minimal structural and interfacial degradation during high‐rate cycling. The initial discharge capacities are 151 mAh g^−1^ for the DAEL and 135 mAh g^−1^ for SAEL, underscoring the improved kinetic performance afforded by the dual‐additive formulation. Electrochemical impedance spectroscopy provides additional insight into interfacial durability (Figure 7d). The improved EIS response of the DAEL system is closely associated with the formation of a more stable interphase, as supported by the smoother and more uniform surface morphology observed in AFM analysis (Figure S16). Such stabilized interfaces effectively suppress impedance buildup during cycling, thereby enhancing the electrochemical performance [66]. After 50 cycles, the DAEL exhibits the lowest interfacial resistance among the three systems, indicating the formation of a stable and ionically conductive interphase. A similar trend is already evident after formation (Figure S17), where the Baseline electrolyte shows the highest resistance, reflecting early‐stage interfacial instability and non‐uniform SEI formation. In contrast, cells using the Baseline electrolyte and SAEL exhibit progressively higher impedance growth during cycling, consistent with continuous electrolyte decomposition and heterogeneous interphase development. This impedance evolution trend aligns with the three‐electrode EIS measurements observed after formation (Figure S18), where the DAEL exhibited reduced interfacial resistance at both the Si anode and NCM811 cathode. Post‐mortem characterization of NCM811 cathodes extracted from Si||NCM811 full cells provides direct evidence of CEI evolution. High‐resolution Cs‐corrected transmission electron microscopy (TEM) images (Figure 7e,f and Figure S19) reveal that cathodes cycled in SAEL form a thick (≈ 7–8 nm) and heterogeneous interphase, whereas those cycled with the DAEL exhibit a thinner (≈ 4–6 nm) and more uniform layer [67]. Such a conformal interphase is favorable for maintaining low interfacial resistance, mechanical integrity, and structural stability during prolonged cycling, while suppressing parasitic interfacial reactions. Additional XPS analysis of the cycled NCM811 cathodes further supported the stabilized CEI chemistry in the DAEL system, revealing the effective suppression of LiPF_6_‐derived interfacial decomposition and noticeable reduction in parasitic reactions on the cathode surface (Figure S20). In contrast, the irregular interphase observed in SAEL is likely to impede Li^+^ transport and increase susceptibility to interfacial fracture, contributing to accelerated capacity fading. Inductively coupled plasma–mass spectrometry analysis (ICP‐MS) after 50 cycles quantifies TM dissolution from the NCM811 cathode in Si||NCM811 full cells (Figure 7g). Ni is the predominant dissolved species in all systems, consistent with its higher mobility and surface reactivity [67, 68]. The total TM dissolution is lowest for the DAEL (54 × 10^4^ ppm), compared to the Baseline electrolyte (59 × 10^4^ ppm) and SAEL (77 × 10^4^ ppm). Dissolution trends for individual metals follow a similar pattern, with Ni dissolution dominating across all systems. The markedly reduced Ni dissolution in the DAEL highlights the effectiveness of the formulation in suppressing interfacial acidity and acid‐driven cathode corrosion [45, 69]. Reduced TM leaching also minimizes metal deposition on the silicon anode, thereby limiting parasitic SEI growth and suppressing charge‐transfer resistance buildup. These effects help preserve the layered structure of NCM811, inhibit rock‐salt phase reconstruction, and ultimately translate into improved Coulombic efficiency, reduced gas evolution, and enhanced long‐term cell stability [61]. The low impedance growth observed in Si||Si symmetric cells assembled using silicon anodes retrieved from Si||NCM811 full cells after 10 cycles further supports this interfacial stabilization Consistent with these results, the DAEL system also exhibited a suppressed open‐circuit voltage decay during self‐discharge testing of Si||NCM811 full cells, indicating reduced parasitic reactions during storage (Figure S21). In contrast to the known instability of SEI and CEI layers in FEC‐containing electrolytes—particularly under fully lithiated and high‐temperature storage conditions—the DAEL effectively suppresses impedance rise during storage at 60°C, indicating the formation of a robust and thermally stable interphase (Figure 7h) [67].

## 3 Conclusion

Overall, this work clarifies that the instability of practical Si||NCM811 full cells originates from coupled interfacial degradation processes, including repeated SEI fracture and continuous electrolyte consumption on the silicon anode, together with surface reconstruction and TM dissolution at the Ni‐rich cathode. These degradation pathways are further accelerated by LiPF_6_‐derived HF and reactive fluorophosphate species. While FEC effectively promotes LiF‐rich SEI formation on silicon, it is insufficient in full‐cell configurations, as acid‐driven electrolyte decomposition and cathode‐side instability persist under high‐voltage and high‐temperature conditions. By introducing DMDMS as a complementary component within a DAEL formulation, HF scavenging and PF_5_ stabilization are simultaneously achieved, enabling effective suppression of parasitic interfacial reactions and concurrent stabilization of both SEI and CEI. As a result, the DAEL delivers improved cycling stability, higher Coulombic efficiency, reduced H_2_ evolution, suppressed electrode swelling, and enhanced rate capability in Si||NCM811 full cells. These findings underscore the importance of electrolyte formulation strategies that account for coupled anode–cathode interfacial chemistry in practical full‐cell systems and demonstrate that synergistic additive interactions provide an effective pathway toward high‐energy‐density and durable lithium‐ion batteries.

## 4 Experimental Section/Methods

### 4.1 Fabrication of Electrodes and Electrolytes

The cathode was prepared using 96.5 wt.% LiNi_0.8_Co_0.1_Mn_0.1_O_2_ (NCM811, EcoPro BM) as the active material, 1.5 wt.% Super P as the conductive agent, and 2 wt.% polyvinylidene fluoride (PVDF, Kureha) as the binder. The anode consisted of 80 wt.% Si–Fe alloy (MKE), 10 wt.% Super P, and 10 wt.% poly(acrylic acid) (PAA, Sigma–Aldrich) as the binder. The physicochemical properties of the Si anode material used in this study were provided by the manufacturer (MKE) and are summarized as follows. The composition of the Si‐based material consists of 80.16 ± 0.5 at% Si and 15.88 ± 0.5 at% Fe, with the remaining 3.96 ± 0.5 at% attributed to minor components. The particle size distribution was characterized by laser diffraction, yielding *D*_10_, *D*_50_, and *D*_90_ values of 1.73, 3.58, and 6.75 µm, respectively, indicating a relatively narrow and controlled size distribution. The tap density of the material was measured to be 1.36 ± 0.05 g mL^−1^, suggesting reasonably dense packing behavior suitable for electrode fabrication. In addition, the specific surface area determined by BET analysis was 6.65 m^2^ g^−1^, which falls within a typical range for Si‐based anode materials and is beneficial for balancing electrochemical reactivity and interfacial stability. These features confirm that the Si anode material employed in this work is well‐defined and suitable for reliable electrochemical evaluation. Moisture contents of both electrodes were measured before and after vacuum drying at 120°C for 12 h. The areal mass loadings of the cathode and anode were 7.57 and 2.1 mg cm^−2^, respectively. The Si anode was prepared with a mass loading of ∼2.1 mg cm^−2^, corresponding to a coating thickness of approximately 13.4 µm. Based on these values, the electrode density (ρ_electrode_) was calculated using the following equation:

$$\rho_{electrode}=\frac{massloading}{electrodethickness}$$

which yielded a value of approximately 1.4–1.5 g cm^−3^. The porosity was determined by:

$$Porosity\;\left(\%\right)=\left(1-\frac{\rho_{electrode}}{\rho_{theoretical}}\right)\times 100$$

where ρ_theoretical_ was calculated from the weighted densities of the individual electrode components (Si–Fe alloy, Super P, and PAA binder). Consequently, the porosity of the Si‐based composite electrode was estimated to be approximately 35%–40%. The Baseline electrolyte (Baseline) comprised 1.15 M LiPF_6_ in EC:DMC:EMC = 2:4:4 vol% (SoulBrain), with moisture and HF contents of 10.7 and 63.7 ppm, respectively. The single‐additive electrolyte (SAEL) was formulated by adding 10 wt.% fluoroethylene carbonate (FEC), and the dual‐additive electrolyte (DAEL) included both 10 wt.% FEC and 4 wt.% dimethoxydimethylsilane (DMDMS, Sigma–Aldrich, 99%) to the Baseline. The viscosities of the electrolytes were measured to be 4.65, 25.6, and 38.6 cP for Baseline, SAEL, and DAEL, respectively.

### 4.2 Electrochemical Measurements

Electrochemical tests were performed using 2032 coin‐type cells assembled in an Ar‐filled glove box (O_2_, H_2_O < 1 ppm) using a polyethylene (PE) separator (SB16C, Asahi Kasei). NCM811 half‐cells (2.3 mAh) were cycled from 3.0 to 4.3 V with two formation cycles at 0.1C followed by cycling at 0.5C. Si half‐cells (3.8 mAh) were cycled from 0.01 to 1.5 V with 0.1C and 0.2C for formation cycles and 0.5C for cycling. Full cells (Si||NCM811, ∼2.77 mAh) with a negative‐to‐positive (N/P) capacity ratio of 1.2 were pre‐cycled at 0.1C and 0.2C, followed by continuous cycling at 0.5C from 2.5 to 4.2 V using a battery tester (WonATech WBCS 3000) at 25°C. Electrolyte volumes of 75 µL for the NCM811 half‐cell and 150 µL for both the Si half‐cell and Si||NCM811 full cell were used. Rate performance was evaluated on Si||NCM811 full cells under cycling at 0.1C, 0.2C, 0.5C, 1C, 2C, 3C, 5C and 10C, followed by a return to 0.1C. To evaluate electrolyte oxidative stability, linear sweep voltammetry (1 mV s^−1^) and cyclic voltammetry (CV, 0.1 mV s^−1^, 0.01–2.0 V) were conducted using WonATech WBCS 3000. Symmetric Si||Si cells were fabricated using fully lithiated Si anodes extracted from full cells after 10 formation cycles at 100% Stage of Charge (SOC), then stored at 60°C for 8 days. Daily impedance measurements were conducted at room temperature using a VMP3 potentiostat (Bio‐Logic).

### 4.3 Characterization

The molecular orbital energy calculations were per‐formed using the B3LYP hybrid density functional conducted in the Spartan`24 program package. The 6–311+G(2d, p) basis sets were used for all the atoms. Ionic conductivity was measured using a SevenExcellence conductivity meter (Mettler Toledo) calibrated with 12.88 mS cm^−1^ standard. HF scavenging capability of DMDMS was evaluated using ^19^Fluorine‐Nuclear Magnetic Resonance (^19^F‐NMR) (Bruker AVANCE NEO 500 MHz) after 7 days storage at 25°C. Chloroform‐d (Sigma–Aldrich) served as internal reference. Contact angles were measured using a Phoenix 300 goniometer (SEO) with a 27‐gauge needle. For postmortem analysis, cycled cathodes were rinsed with dimethyl carbonate (DMC) for 15 min and dried in an Ar‐filled glove box. Field‐Emission Scanning Electron Microscope (FE‐SEM) (TESCAN MAGNA, 10 kV) was used to observe Si–Fe anodes after one cycle at 25°C. SEI composition was analyzed using XPS (ESCA, Al Kα = 1486.6 eV). Atomic Force Microscopy (AFM) (Dimension Icon NanoScope IV) was conducted over a 5 × 5 µm^2^ scan area with ± 500 nm height range. Time‐of‐Flight Secondary Ion Mass Spectrometry (TOF‐SIMS) (M6, IONTOF GmbH) analysis was conducted under Ultra‐high vacuum (UHV) using Bi_3_^+^ ion source (30 keV, ∼0.1 pA) and Cs^+^ sputtering (2 keV, ∼100 nA) for 5077 s over 150 × 150 µm^2^ area. Inductively Coupled Plasma Mass Spectrometry (ICP‐MS) (Agilent 7700S) quantified transition metal dissolution from NCM811 cathodes. Samples were rinsed, dried, and digested in 7 mL of 70% HNO_3_ + 3 mL of 35% HCl at 200°C for 30 min using a microwave digestion system. For high‐resolution imaging, cleaned cathodes were mounted on Transmission Electron Microscopy (TEM) grids and analyzed using Cs‐Corrected Transmission Electron Microscopy (Cs‐TEM) (Spectra 300, ThermoFisher) at 20–200 keV and 0.26 nm resolution. In situ Differential Electrochemical Mass Spectrometry (DEMS) was installed by a home‐built design to monitor gas evolution during galvanostatic charging of NCM811||Li half‐cells at 0.3C and 25°C. The cathode materials were cast on a SUS mesh and used as a working electrode. A coin‐type cell with a meshed top was assembled with the working electrode, a lithium counter electrode, and a separator soaked with the electrolyte. The coin cell was placed inside a cell holder, which had two capillaries for a gas to flux in and to flow out of the cell. The gases generated from the cell were accumulated in a headspace of the cell for a programmed time (typically 10 min) and then swept by a carrier gas argon (Ar) to a mass spectrometer (UGA‐200, Stanford Research Systems). The gases were identified by a mass‐to‐charge ratio (m/z = 2 for H_2_) and recorded as a partial pressure (Torr).

## Funding

Nano‐Materials Technology Development Program through the National Research Foundation of Korea (NRF) funded by Ministry of Science and ICT (Grant No. NRF‐2021M3H4A3A02086208), the Materials and Part Technology Development Program (No. RS‐2024‐00432014) funded by the Ministry of Trade, Industry and Energy (MOTIE) of Korea, National Research Council of Science & Technology (NST) grant by the Korea Government (MSIT) (No. GTL24011–000), and the Korea Research Institute of Chemical Technology (KRICT) (No. KS2622‐40).

## Conflicts of Interest

The authors declare no conflicts of interest.

## Supporting information




**Supporting File**: advs76056‐sup‐0001‐SuppMat.docx.

## Data Availability

The data that support the findings of this study are available from the corresponding author upon reasonable request.
