# Supplementary material for: Electrolyte Design for Simultaneous Interfacial Stabilization in Si||NCM811 Full Cells
Source: Adv Sci (Weinh). 2026 Jun 9:e76056. Online ahead of print. doi: 10.1002/advs.76056 (PMC13336446; doi:10.1002/advs.76056)

Supporting Information

Electrolyte Design for Simultaneous Interfacial Stabilization in Si||NCM811 Full Cells

Seo Yun Jang, Kyu Hong Lee, Juhyoung Kim, Dong Wook Kim, Kyu Hyoung Lee, Do Youb Kim, and Se-Hee Kim*

S. Y. Jang, K. H. Lee, J. Kim, Dr. D. W. Kim, Dr. D. Y. Kim, Dr. S. -H. Kim

Advanced Battery Research Center

Korea Research Institute of Chemical Technology (KRICT)

141 Gajeong-ro, Yuseong-gu, Daejeon, 34114, Republic of Korea

E-mail: [seheekim@krict.re.kr](file:///D:\NCM%20Si%20full%20cell%20electrolyte\Advanced%20Energy%20Materials\seheekim@krict.re.kr)

K. H. Lee

Department of Chemical and Biomolecular Engineering

Yonsei University

50 Yonsei-ro, Seodaemun-gu, Seoul, 03722, Republic of Korea

J. Kim, Prof. K. H. Lee

Department of Materials Science and Engineering

Yonsei University

50 Yonsei-ro, Seodaemun-gu, Seoul, 03722, Republic of Korea

Dr. D. W. Kim

Department of Advanced Materials and Chemical Engineering

University of Science and Technology (UST)

217 Gajeong-ro, Yuseong-gu, Daejeon, 34113, Republic of Korea

**Table S1.** AFM roughness parameters of the Si electrode (a) after formation (b) after 50 cycles.


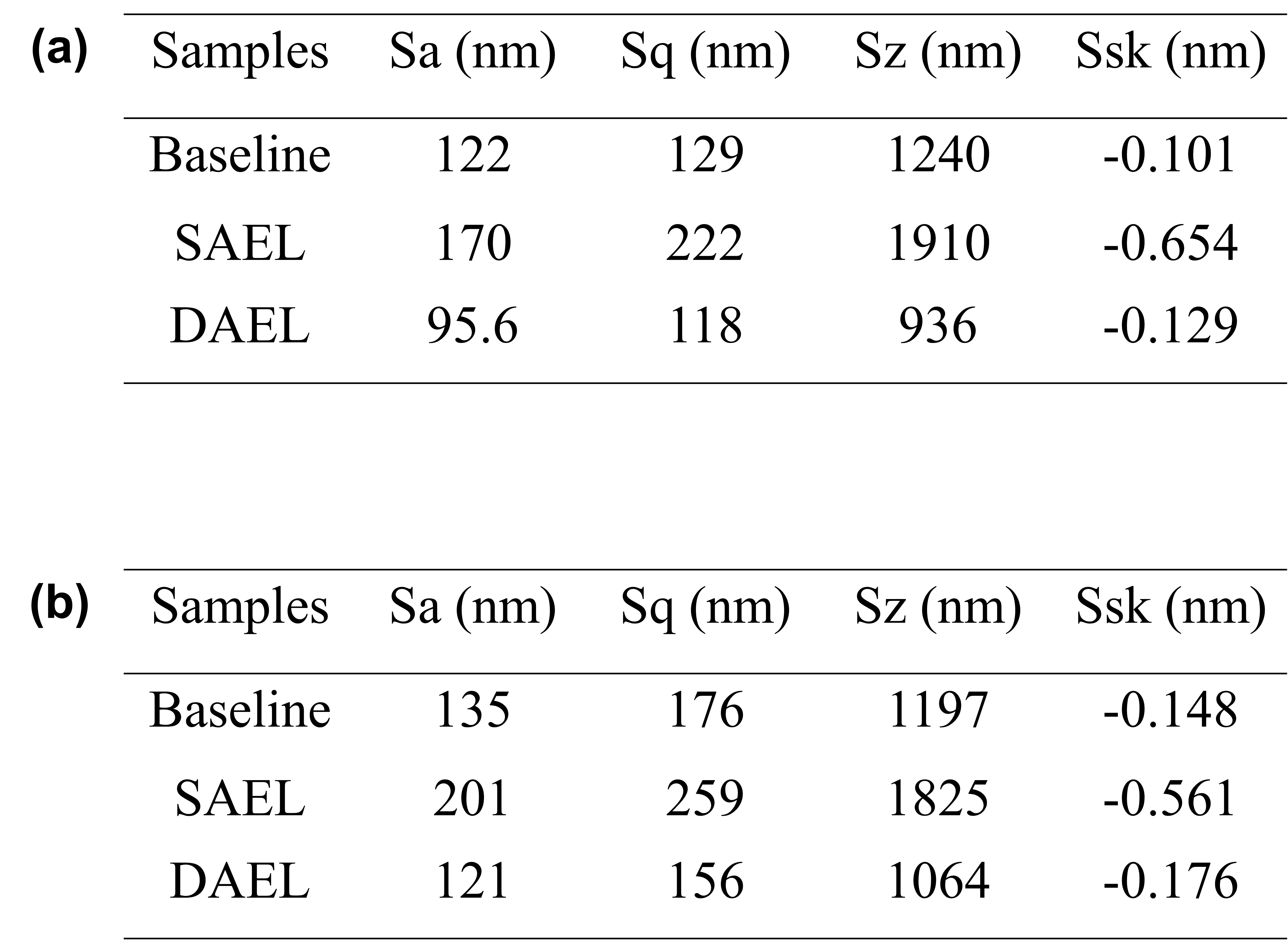


**Figure S1.** Cyclic voltammetry (CV) profiles of Li||SUS cells with (a) Baseline (b) SAEL and (c) DAEL.


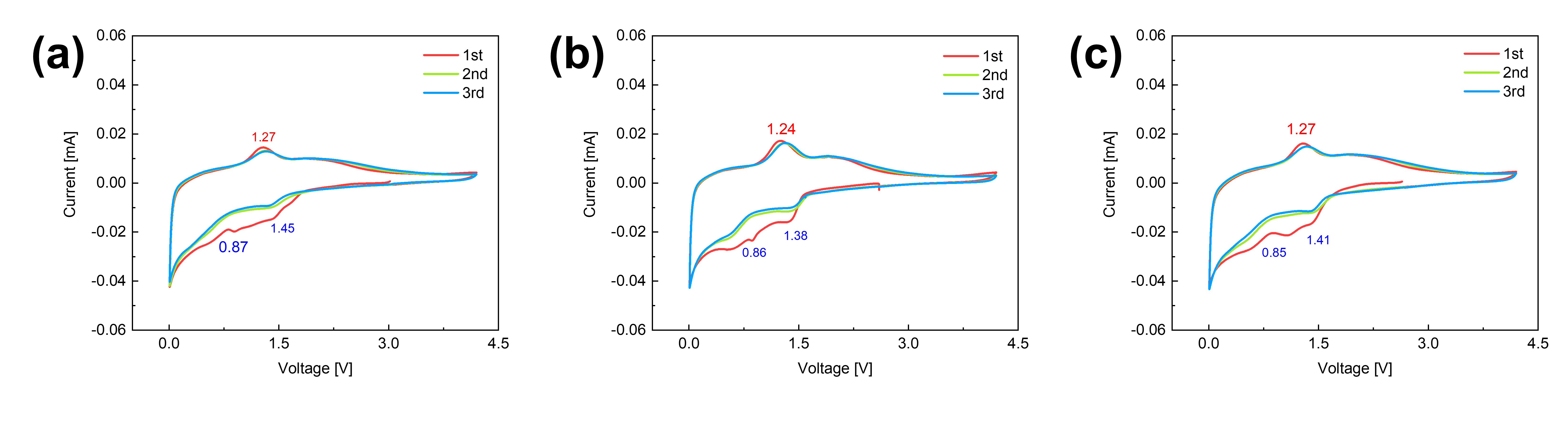


**Figure S2.** Ionic conductivity of various electrolytes, including Baseline, FEC-containing, and DMDMS + FEC mixed electrolytes.

**Figure S3.** Contact angle measurements of electrolytes on the separator surface: (a) Baseline (b) SAEL and (c) DAEL.


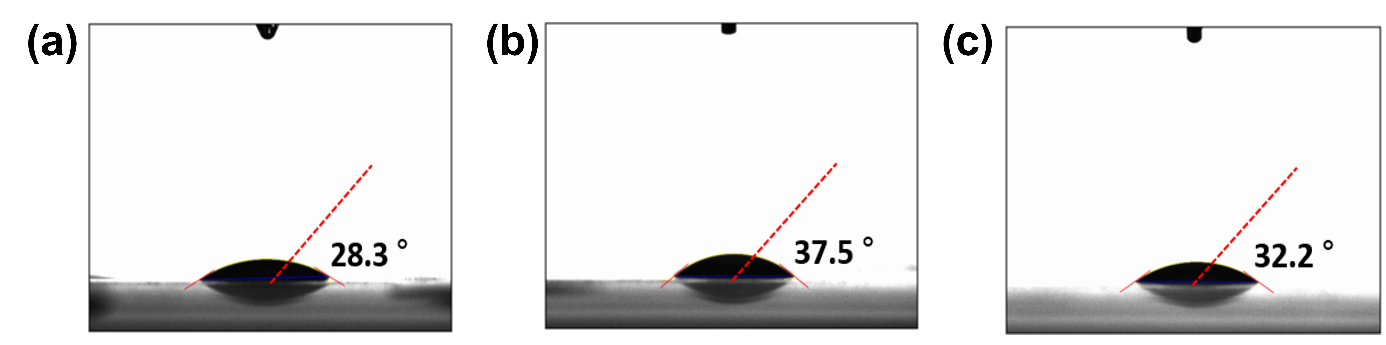


**Figure S4.** Thermal stability and discoloration behavior of electrolytes during high-temperature storage. Visual appearance of electrolytes after storage at 60 °C for (a) 5 days and (b) 2 weeks.

**
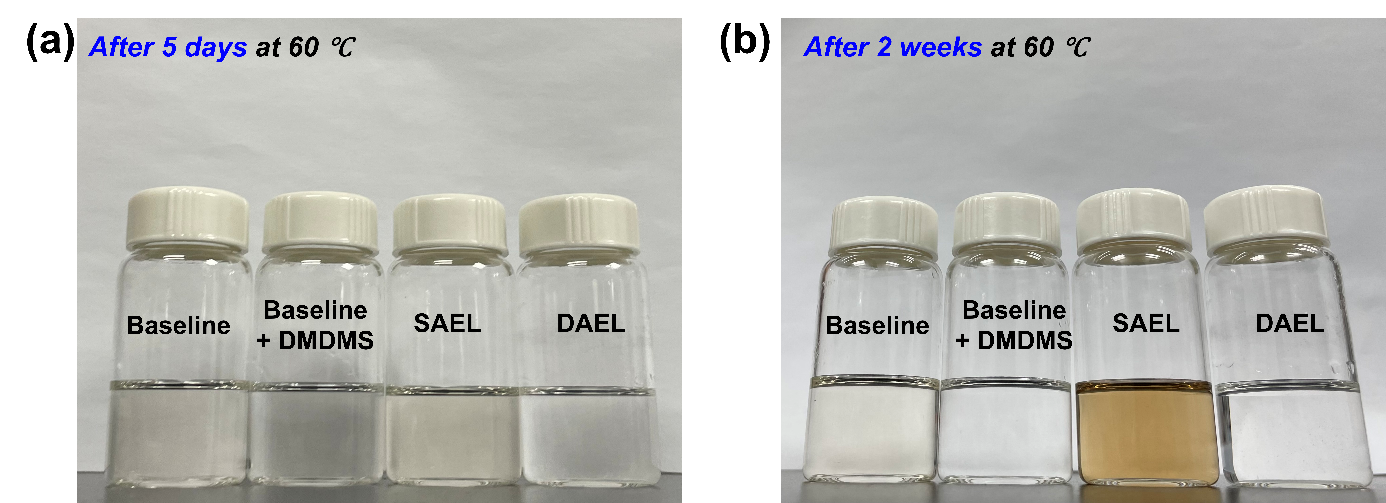
**

**Figure S5.** ^19^F NMR spectrum of the Baseline + DMDMS electrolyte after storage with 500 ppm deionized water.


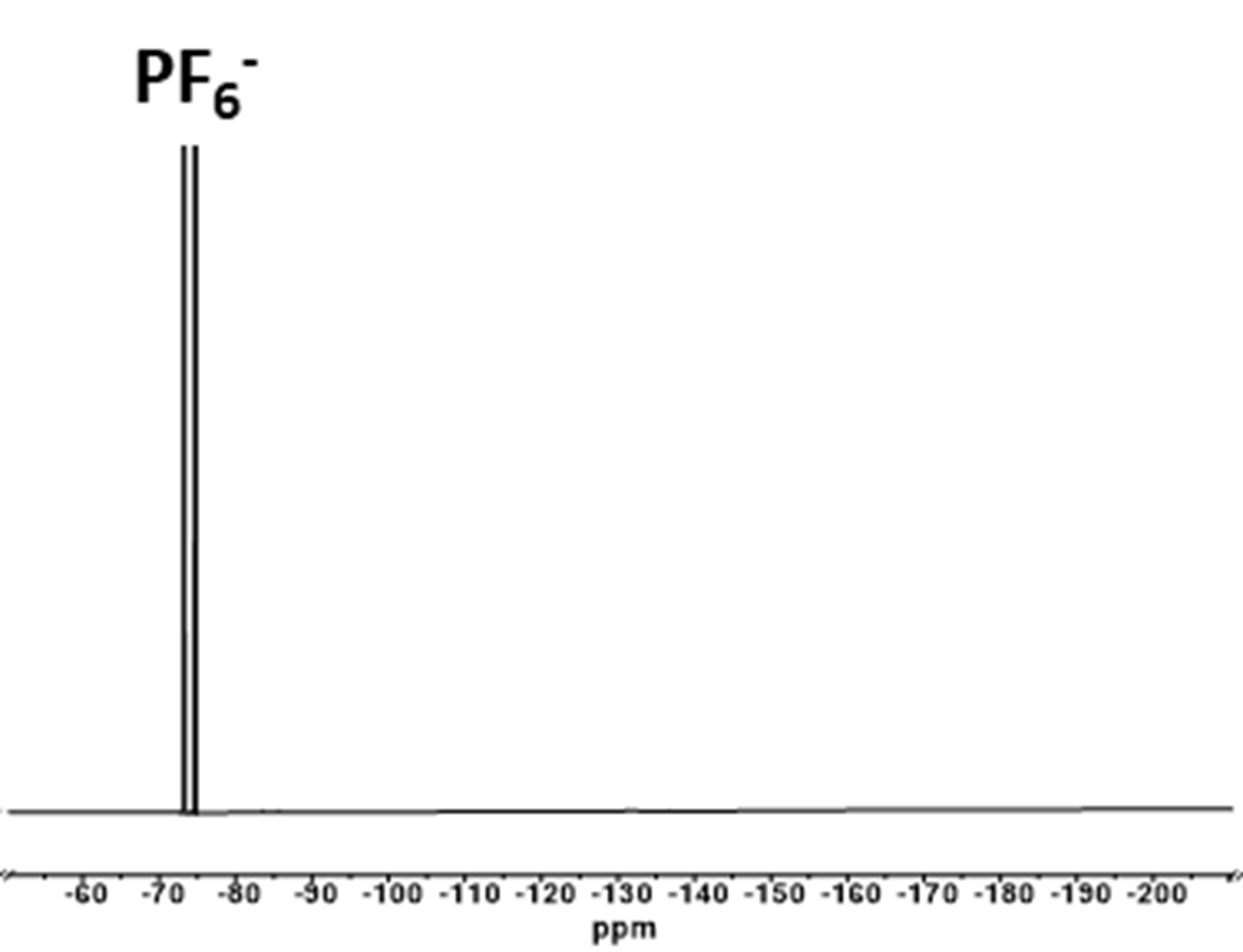


**Figure S6.** Cyclic voltammetry (CV) profiles of Si||Li half-cells using Baseline (a) SAEL (b) and (c) DAEL.


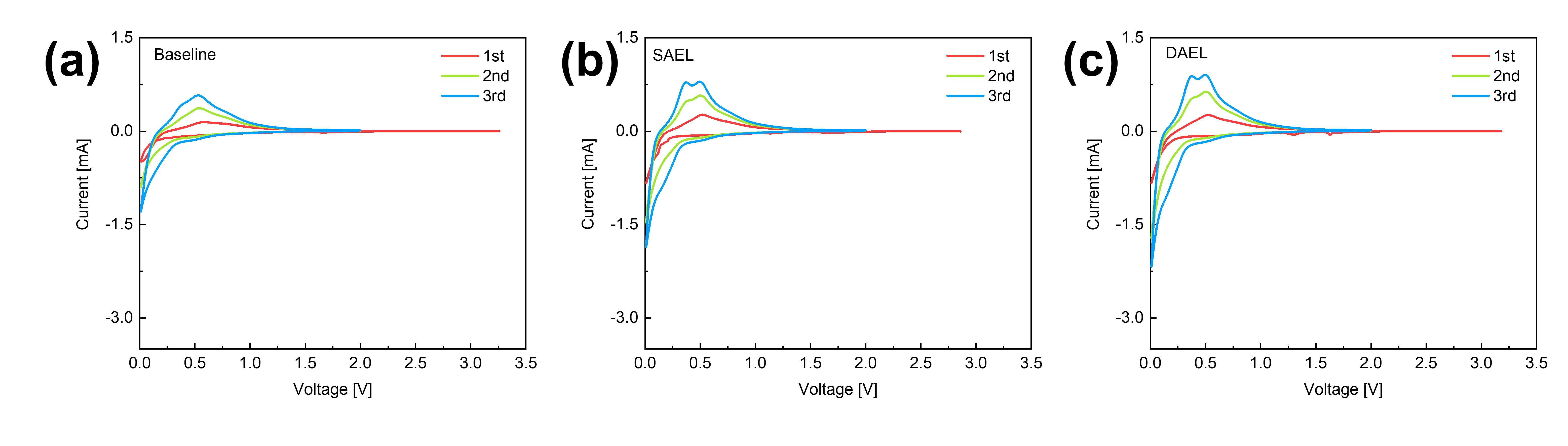


**Figure S7.** EIS spectra of Si||Li half-cells using Baseline, SAEL, and DAEL measured after (a) formation (b) 20^th^ (c) 40^th^ (d) 60^th^ and (e) 80th cycles.


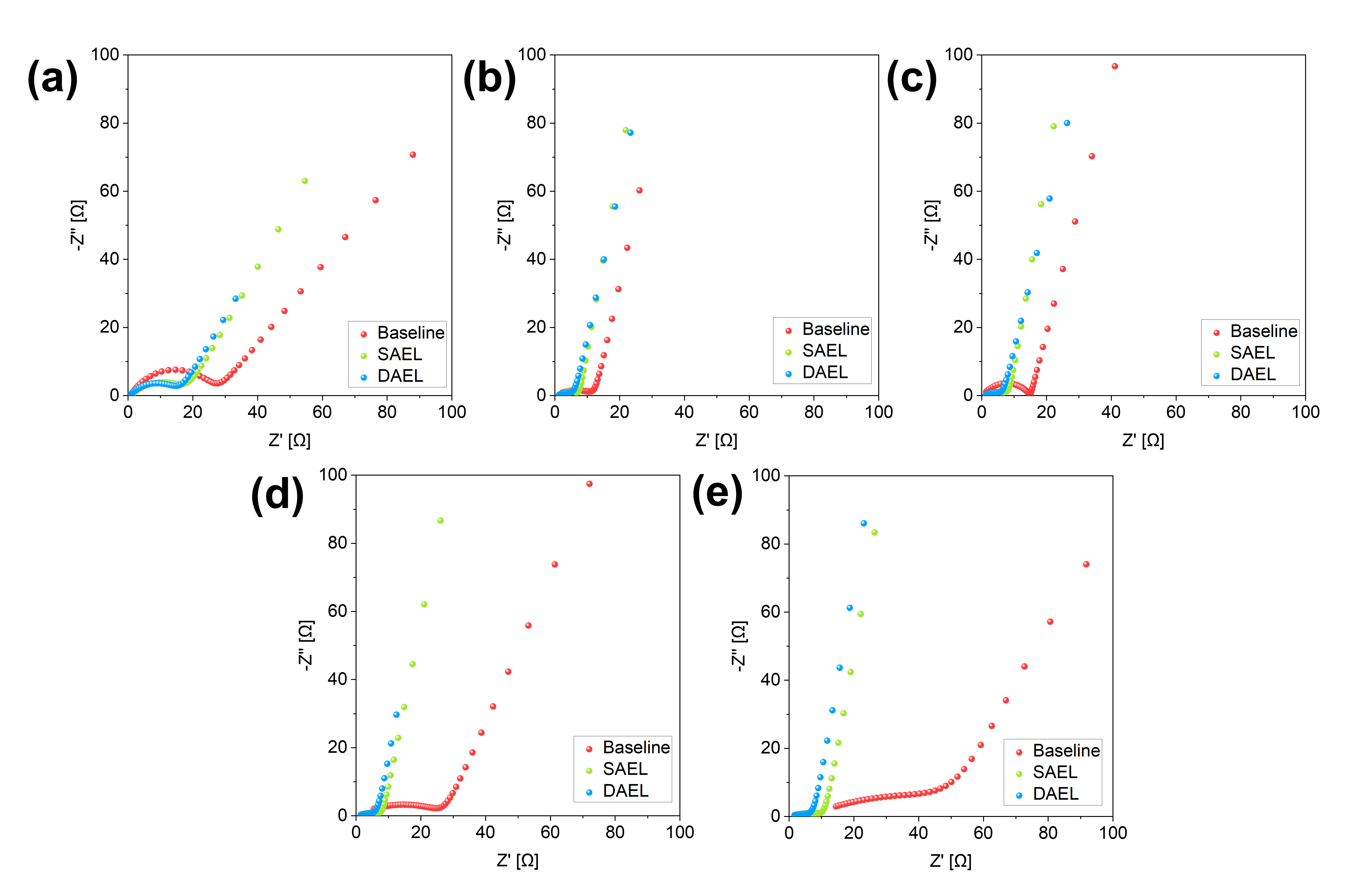


**Figure S8.** Cross-sectional SEM image of the pristine Si composite electrode before cycling. The electrode exhibited a thickness of approximately 13.4 μm.


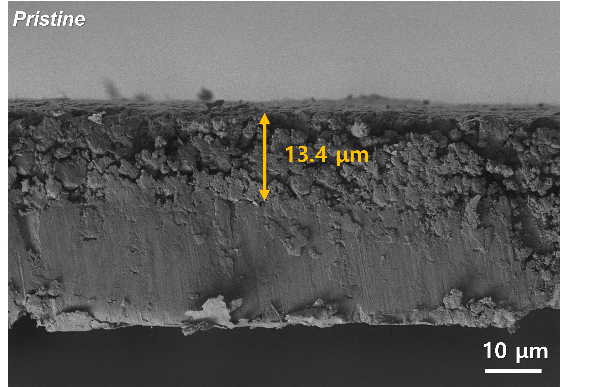


**Figure S9.** Merged TOF-SIMS 3D ion distribution maps of Si electrodes after 50 cycles using (a) Baseline (b) SAEL and (c) DAEL.


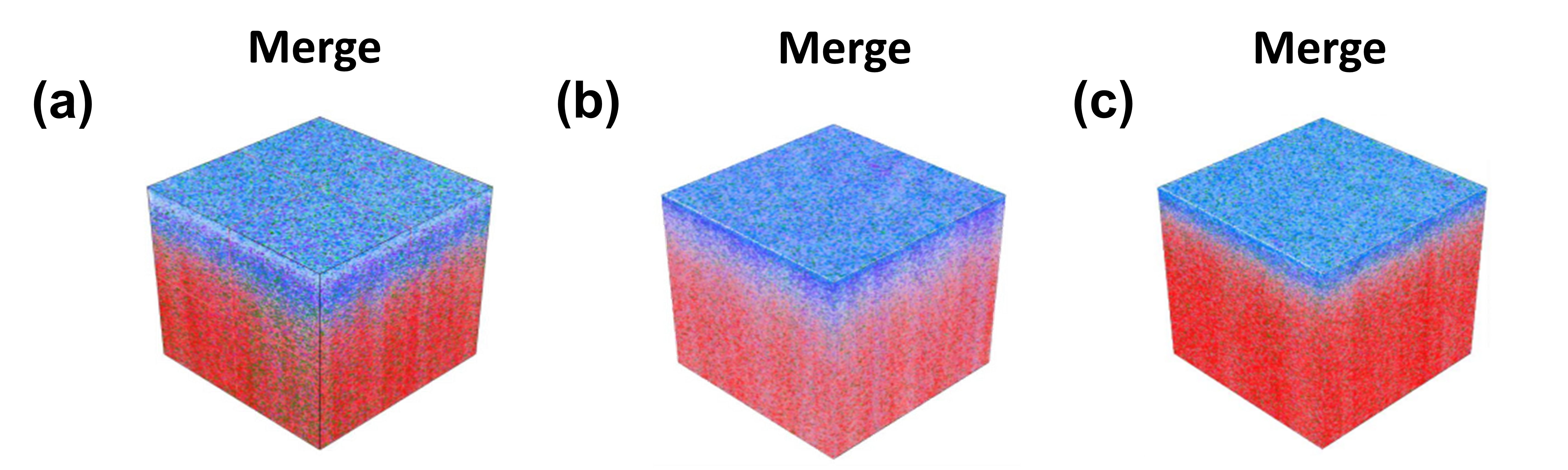


**Figure S10.** TOF-SIMS 3D ion distribution maps of the Si electrode after 50 cycles using the (a) Baseline (b) SAEL showing depth profiles of Si, CHO₂⁻, LiF and PO_2_⁻ species.


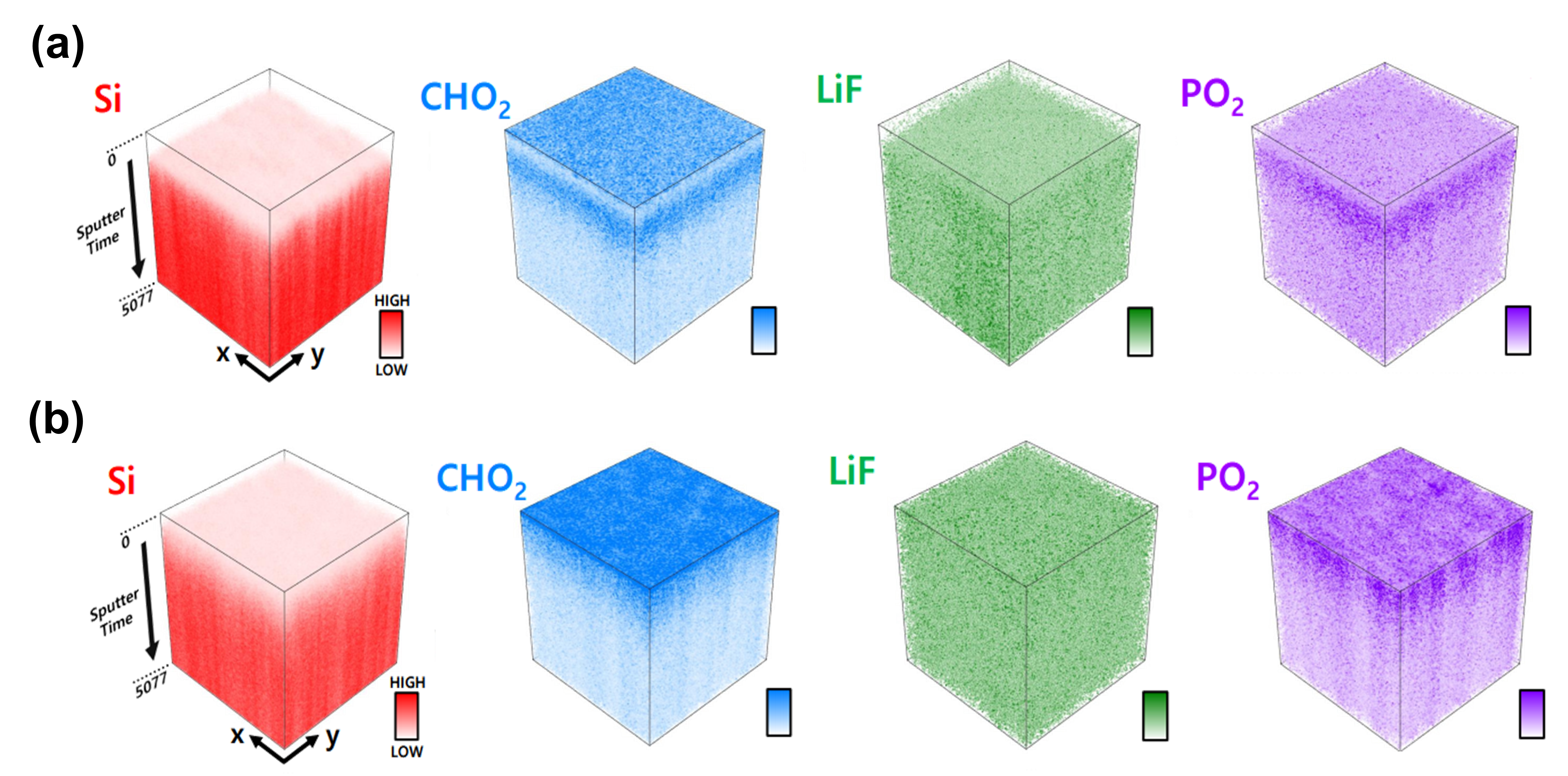


**Figure S11.** SEM images of Si electrodes after formation. (a–c) show surface morphologies, and (d–f) display corresponding cross-sectional views for Baseline, SAEL and DAEL.

**
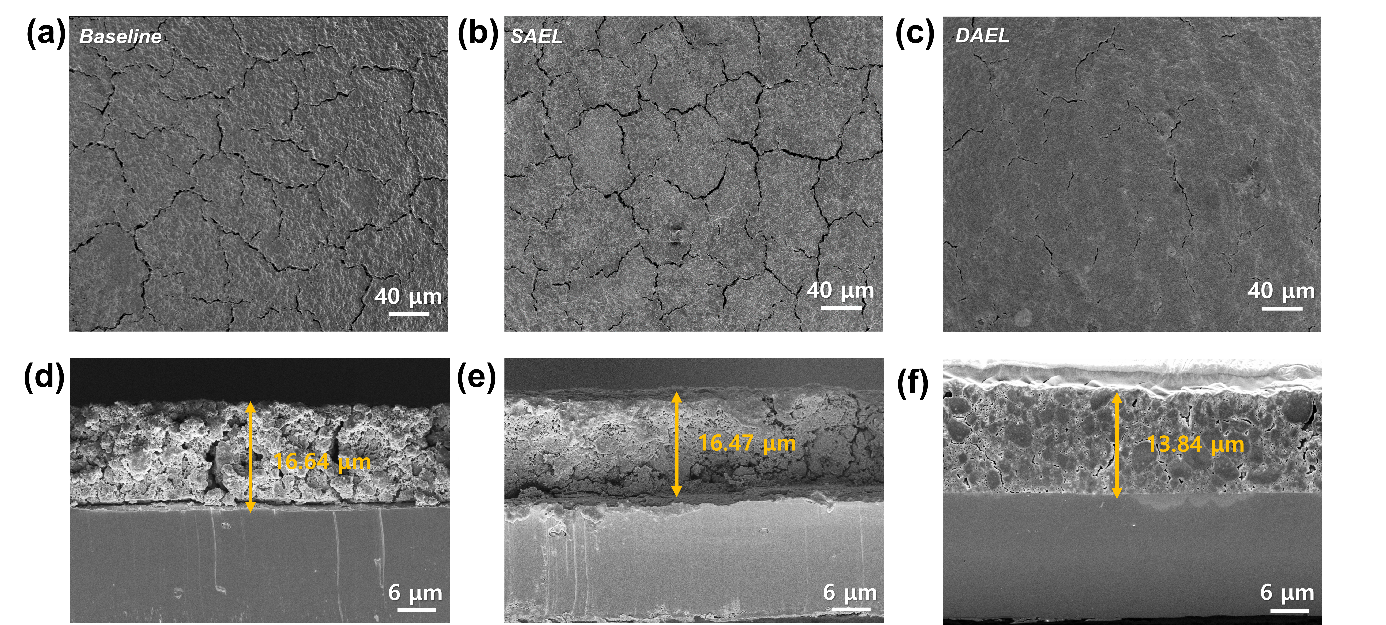
**

**Figure S12.** AFM images of Si electrode surfaces after 50 cycles using (a) Baseline (b) SAEL and (c) DAEL.


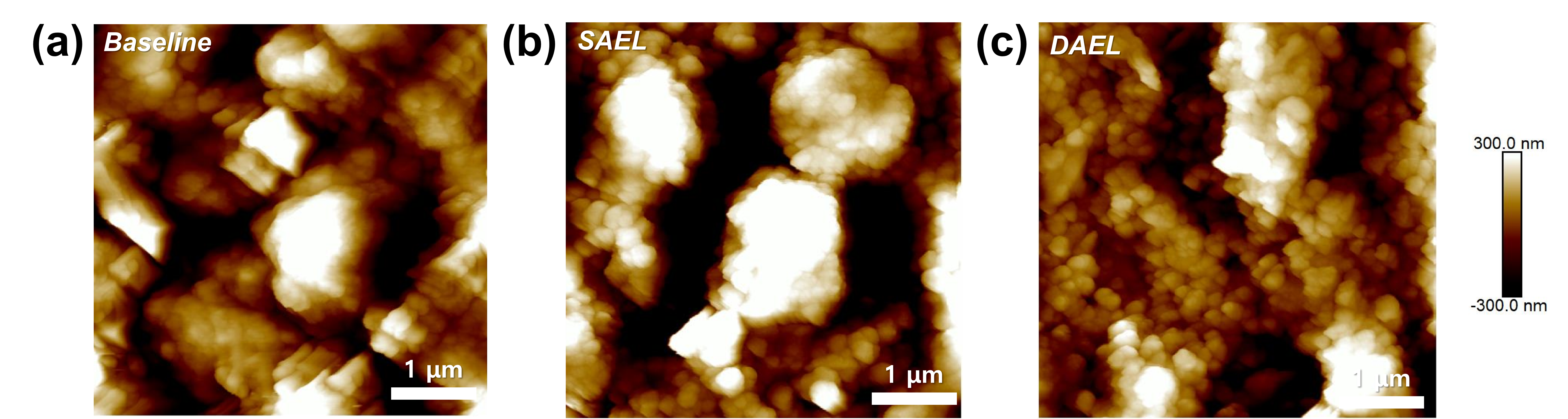


**Figure S13.** AFM surface images of Si electrodes after formation using different electrolytes, Top-view 2D AFM images for (a) Baseline, (b) SAEL, and (c) DAEL, Corresponding 3D AFM topography images for (d) Baseline, (e) SAEL and (f) DAEL.


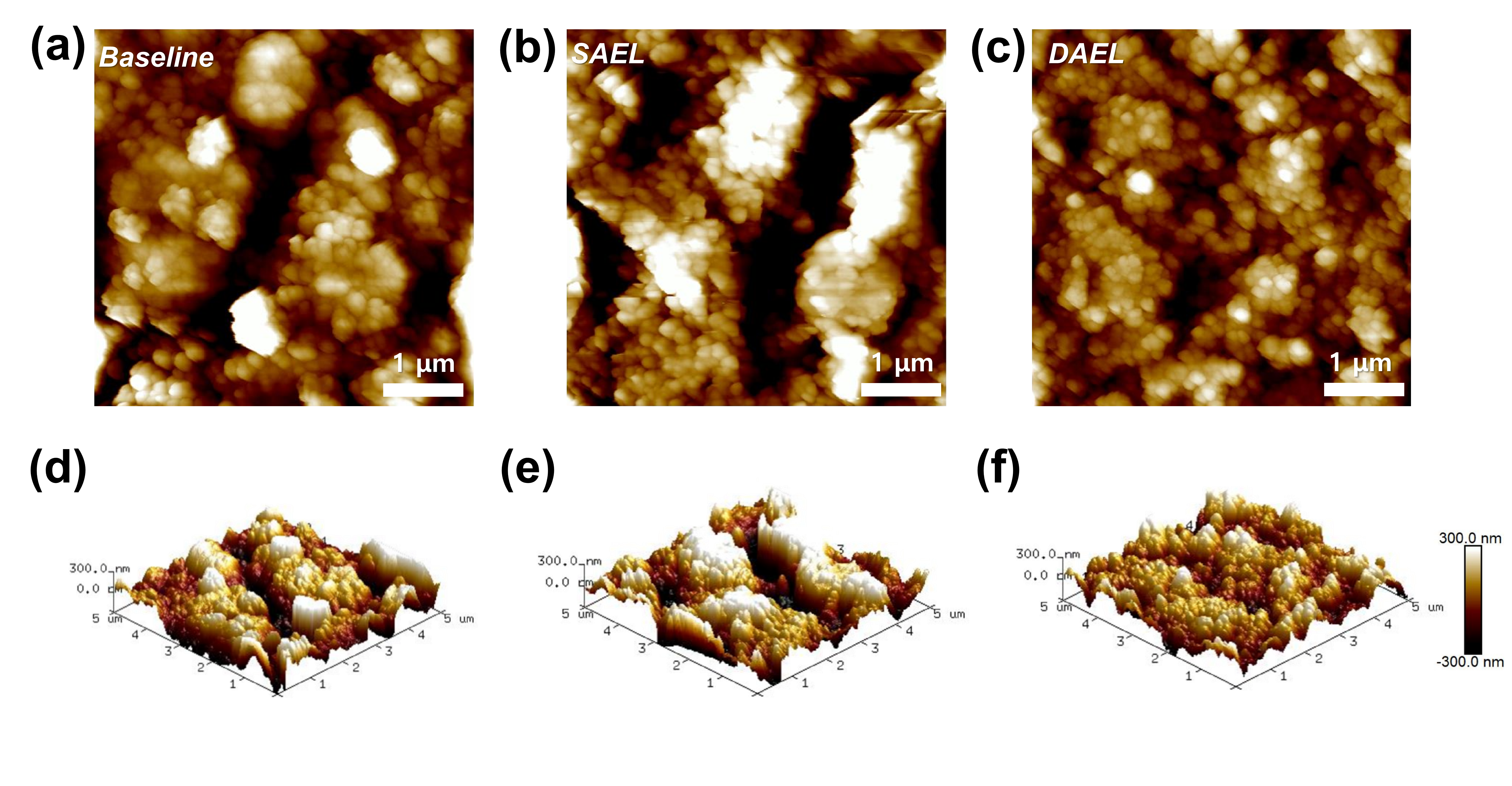


**Figure S14.** Electrochemical performance of Si||NCM811 full cells using Baseline and Baseline + DMDMS electrolytes. (a) Initial charge–discharge voltage profiles and (b) discharge capacity retention.


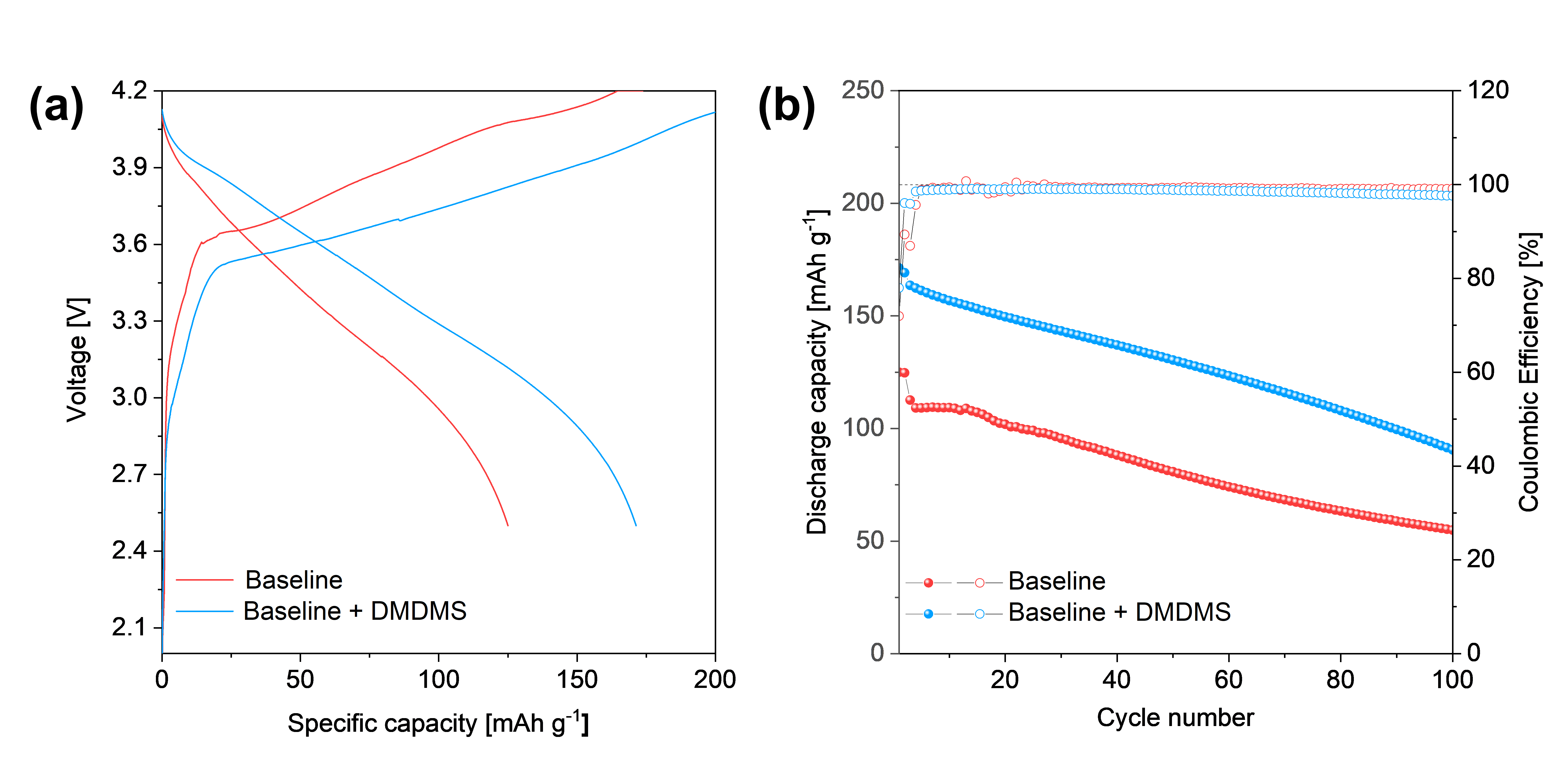


**Figure S15.** SEM images of Si anodes retrieved from Si||NCM811 full cells after cycling with different electrolytes. Electrodes after 30 cycles using (a) Baseline, (b) SAEL, and (c) DAEL, and after 50 cycles using (d) Baseline, (e) SAEL, and (f) DAEL.


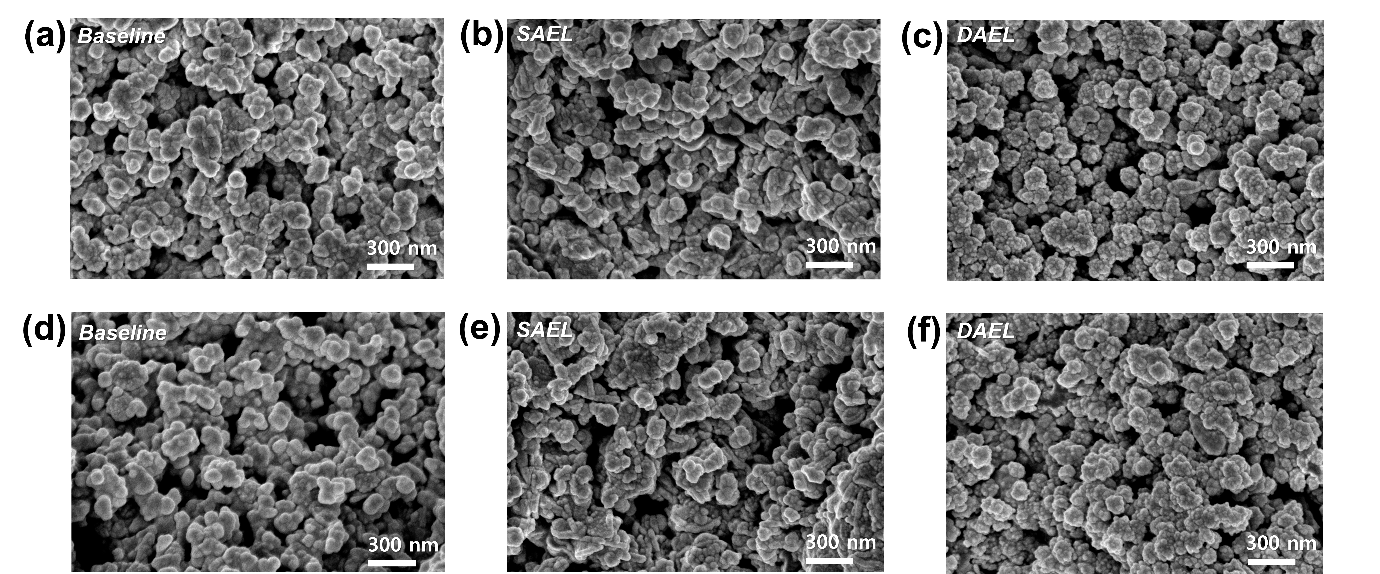


**Figure S16.** AFM surface images of Si electrodes extracted from Si||NCM811 full cells after 30 cycles using different electrolytes. Corresponding 3D AFM topography images for (a) Baseline, (b) SAEL, and (c) DAEL.


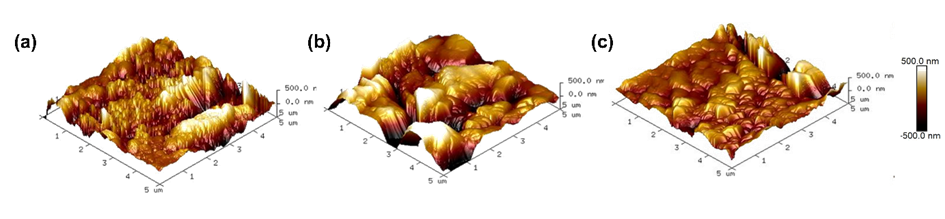


**Figure S17.** EIS spectra after formation in Si||NCM811 full cell.

**Figure S18.** Three-electrode EIS spectra of Si||NCM811 full cells after formation cycling using different electrolyte systems, representing (a) Baseline, (b) SAEL and (c) DAEL.


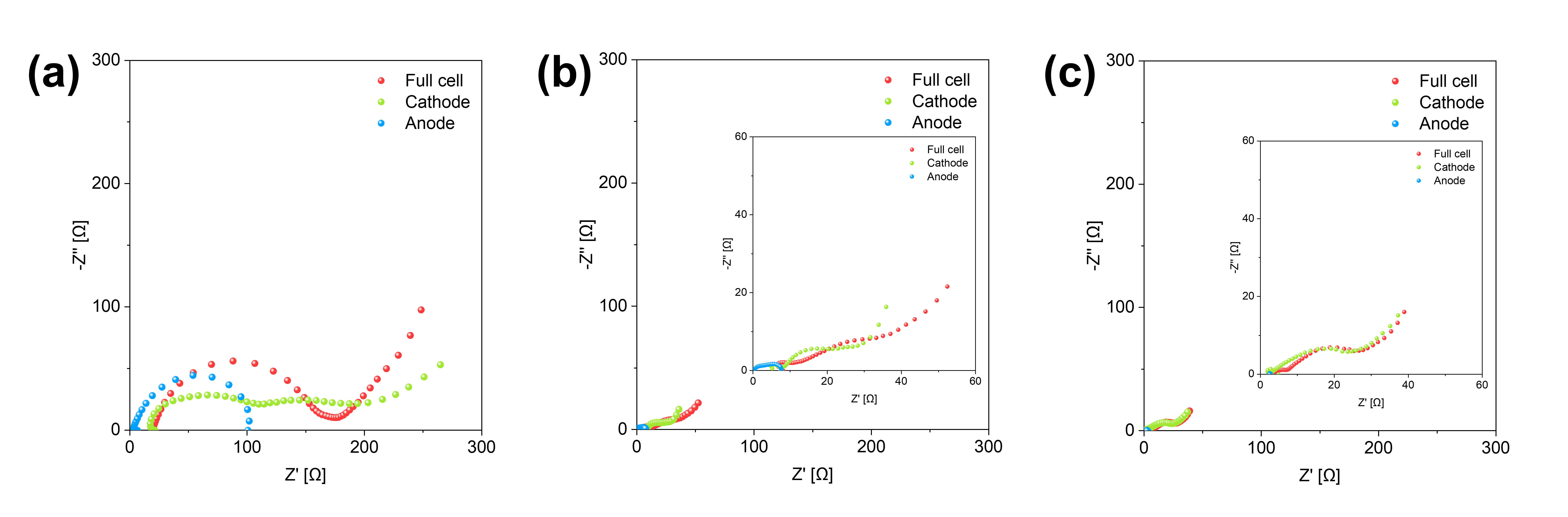


**Figure S19.** TEM images of cathodes extracted from Si**||**NCM811 full cells after 50 cycles, showing the CEI thickness for (a) SAEL and (b) DAEL.


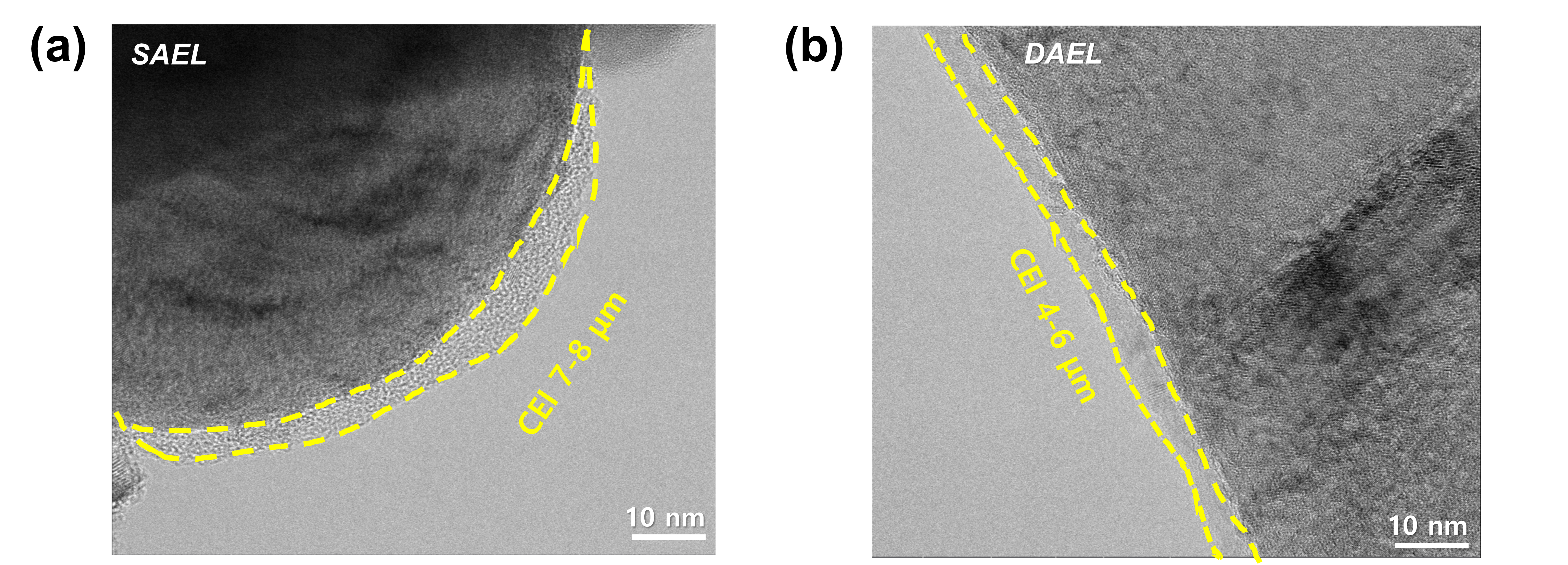


**Figure S20.** XPS spectra of cycled NCM811 cathodes retrieved from Si||NCM811 full cells after 10 cycles, showing the (a) F 1s and (b) O 1s spectra evaluated with Baseline, SAEL and DAEL systems.


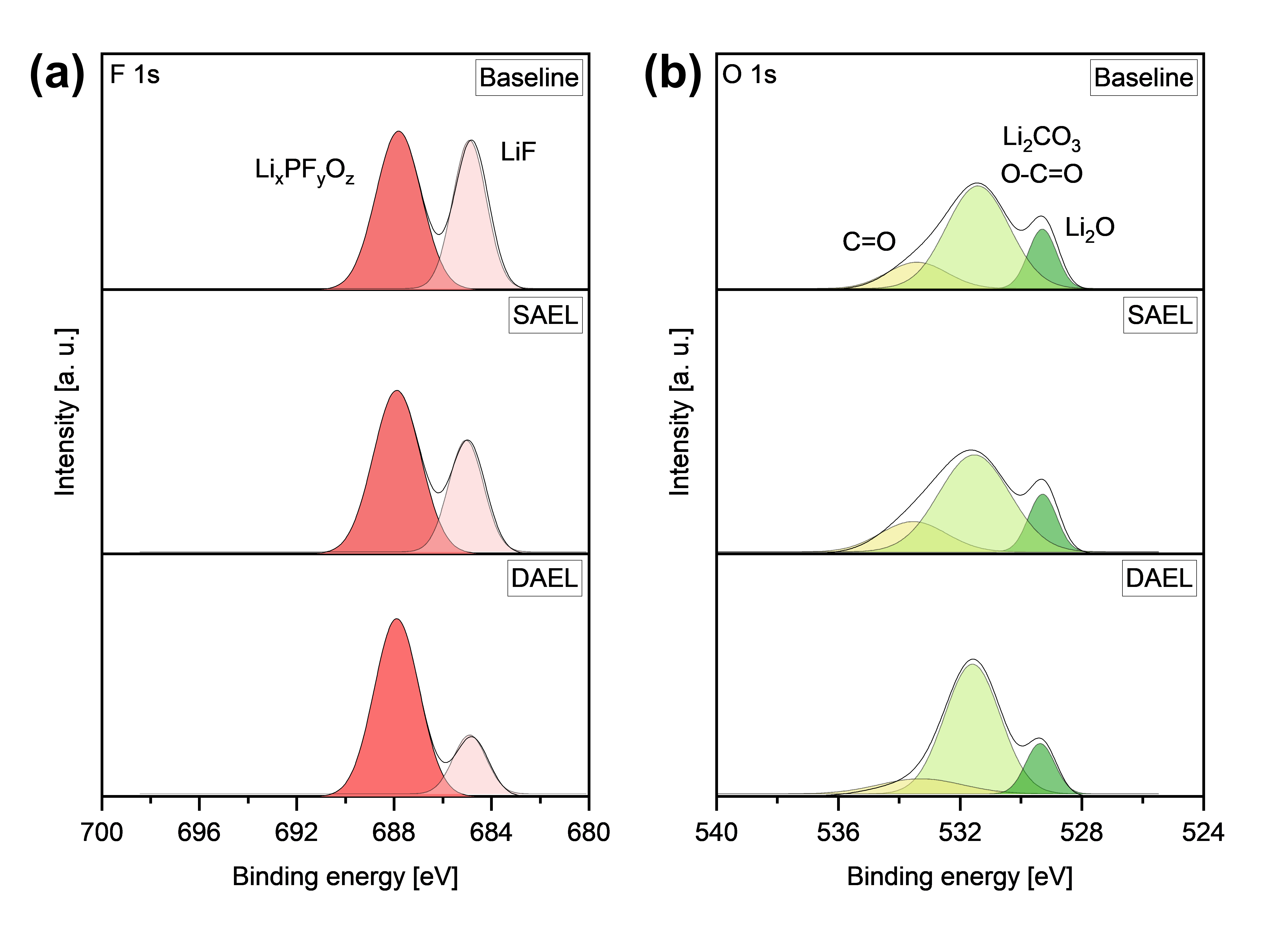


**Figure S21.** Open-circuit voltage decay during self-discharge of Si||NCM811 full cells after charging to a 100% state of charge (SOC) during the formation cycle.


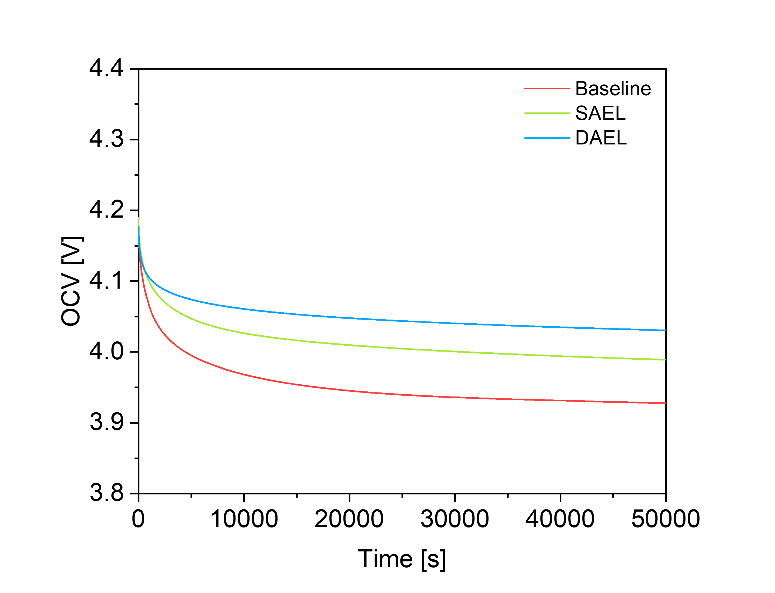

Supplement: Supplementary file 1 — Supporting File: advs76056‐sup‐0001‐SuppMat.docx. [file ADVS-9999-e76056-s001.docx]
